# Enhanced Photocatalytic Proton‐Coupled Electron Transfer by Ligand Design in a Zr Coordination Cage

**DOI:** 10.1002/cssc.202500219

**Published:** 2025-03-19

**Authors:** Pedro J. Jabalera‐Ortiz, Alvaro M. Rodriguez‐Jimenez, Rebecca Vismara, Pedro Delgado, Natalia M. Padial, Jorge A. R. Navarro, Pablo Garrido‐Barros

**Affiliations:** ^1^ Departamento de Química Inorgánica Facultad de Ciencias Universidad de Granada and Unidad de Excelencia en Química (UEQ) Avda. Fuente Nueva s/n 18071 Granada Spain

**Keywords:** Proton-coupled electron transfer, photoredox catalysis, homogeneous catalysis, reaction mechanisms, coordination cages

## Abstract

Developing excited state proton and electron donors is a promising area of research that merges the benefits of proton‐coupled electron transfer (PCET) with the use of light as renewable energy input. Based on the demonstrated PCET reactivity of Zr coordination cages for reductive photocatalysis, here we synthetize and characterize a new cage with enhanced photocatalytic activity. The new design targets the extended biphenyl‐4,4‐dicarboxylate linker with an amino group in the *meta* position relative to the carboxylate. Our results show that these aspects are key to increase the stability and reduction power of the excited state, features that are typically tuned by inductive effects. As a result, the new Zr‐cage promotes significantly faster PCET reactions than the previous related platform, resulting in higher chemical and quantum yields. We further showcase how the solvent can impact the photophysical properties and the PCET reaction rates depending on the cage structure. These results highlight the factors that influence excited state PCET reactivity and complement similar efforts made in the realm of H_2_ evolution.

## Graphical abstract

Ligand size and substitution pattern impact the photophysical and redox properties of a molecular Zr coordination cage by increasing the stability and reduction power of the excited state. These aspects have a positive impact on its photocatalytic PCET reactivity, leading to higher kinetic rates and enhanced quantum and chemical yields for the reduction of acetophenone as model substrate.

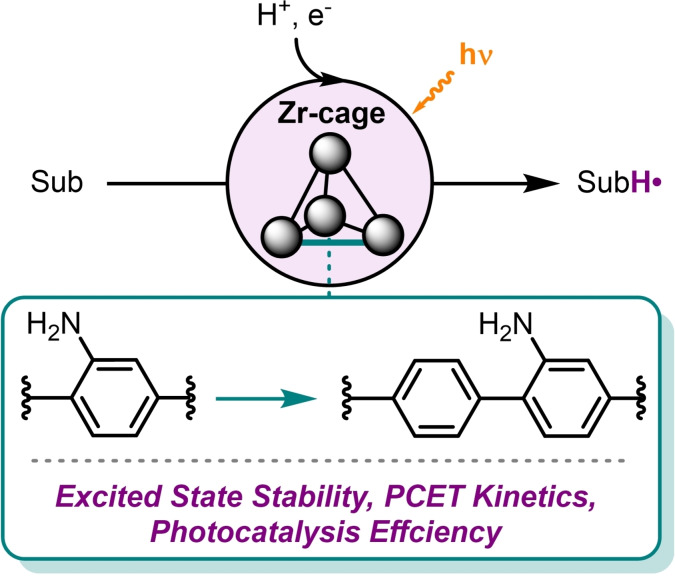

## Introduction

The interplay of photons, protons and electrons is crucial for life and is the basis for the development of solar‐to‐chemicals technologies.[^1^, ^2^] Designing photoredox catalysts has become a prime target to harness the energy of visible light for the synthesis of valuable chemicals and fuels.[^3^, ^4^, ^5^, ^6^] Photons can interact with the catalysts and promote the formation of an excited state (ES) that triggers the transfer of electrons to forge new bonds. However, accessing and controlling highly reactive excited states for a target reaction remains a major challenge.[^7^, ^8^, ^9^]

**Figure 1:**
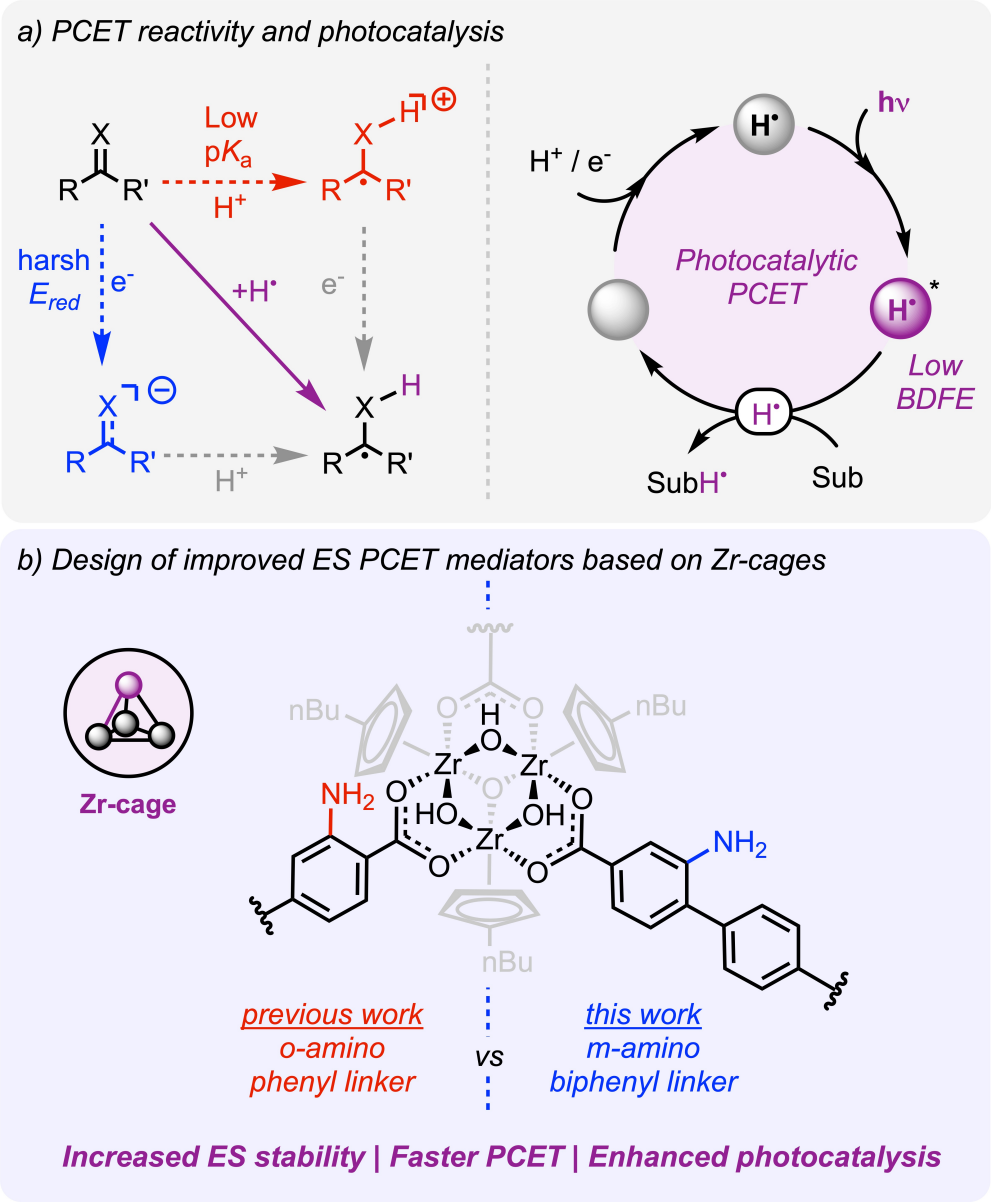
(a) Square mechanism showcasing the PCET reactivity and a reductive PCET photocatalytic cycle. (b) Design of a new Zr coordination cage with enhanced photocatalytic PCET reactivity. ES is excited state and BDFE is bond dissociation free energy.

In this regard, proton‐coupled electron transfer (PCET) mechanisms can offer distinct benefits. The movement of protons along electrons can bypass the formation of high energy‐demanding intermediates and keeps the charge neutrality of the redox process (Figure 1a).[^10^, ^11^] These aspects translate into important mechanistic benefits as they enable challenging redox reactions at milder conditions and higher thermochemical efficiency. Thus, using photons to promote PCET is becoming an increasingly attractive approach in synthetic chemistry.[^12^, ^13^, ^14^, ^15^, ^16^] Important milestones in the field involve the use of Ru and Ir polypyridyl complexes with either pendant (e. g., imidazole ligands) or exogenous acid/base reagents to promote excited state PCET (ES PCET).[^17^, ^18^, ^19^, ^20^, ^21^] However, the development of photocatalytic H^+^ and e^–^ transfer mediators is an ongoing task towards understanding their reactivity and design, and implementing them into synthetic methodologies (Figure 1a).[^13^, ^16^]

We recently demonstrated that a Zr coordination cage featuring a 2‐aminoterephtalate linker, **1‐NH_2_** ^**4+**^ (Figure 1b), is capable to promote ES PCET upon blue light irradiation, disfavoring the competing formation of H_2_.^[22]^ This reactivity was further leveraged for the photocatalytic reduction of different organic substrates. Encouraged by these results and the exciting work from Gascon group showing the potential impact of the amino group position,^[23]^ herein we report a new Zr‐cage, **10‐mNH_2_** ^**4+**^ (Figure 1a), with enhanced photocatalytic PCET reactivity. This new cage with a 2‐amino‐[1,1’‐biphenyl]‐4,4’‐dicarboxylate (BPDC‐m‐NH_2_) ligand provides a more extended π‐system and places the ‐NH_2_ fragment in the *meta* position relative to the carboxylate. This new design increases the stability and reduction power of the corresponding excited state relative to **1‐NH_2_** ^**4+**^, resulting in enhanced PCET reactivity and offering important insights into the design of new molecular photocatalytic PCET mediators for solar‐to‐chemical conversion.

## Results and Discussion

The new Zr‐cage, **10‐mNH_2_** ^**4+**^, was synthetized following a similar procedure to that reported for **1‐NH_2_** ^**4+**^.^[24]^ Briefly, the zirconocene precursor, the 2‐amino‐[1,1’‐biphenyl]‐4,4’‐dicarboxylic acid (BPDC‐*m*‐NH_2_) and water were added to *N,N*‐dimethylformamide (DMF) and the mixture was heated to 60 °C in a sealed flask for 12 h. The reaction proceeds via hydrolysis of the zirconocene with water to form the metal cluster that further coordinates the organic linker. The newly formed cage precipitates from DMF and was washed and isolated providing a crystalline solid suitable for X‐ray diffraction. This compound was also characterized by mass spectrometry, ^1^H NMR and elemental analysis to confirm its formation, and by UV‐vis and IR spectroscopy to extract the spectroscopic signatures (Figure S1–S4).

**Figure 2:**
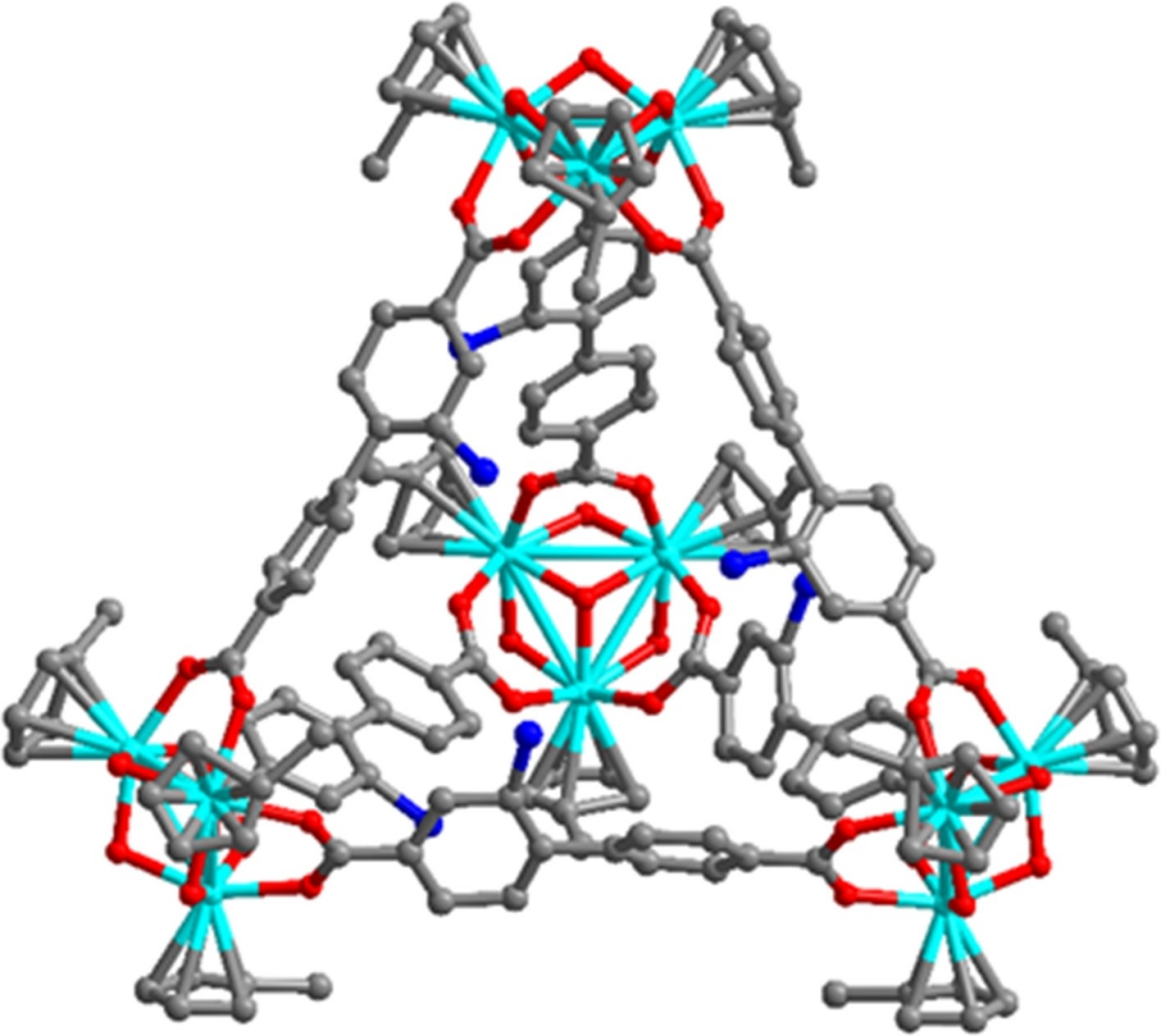
Crystal structure of the tetrahedral cage **10‐mNH_2_**. Color code: zirconium, light blue; carbon, gray; and oxygen, red. Hydrogens, the disordered rings of the ligand and disordered NH_2_ residues are not depicted for clarity.

The crystal and molecular structure of **10‐mNH_2_** ^**4+**^ has been univocally established using single‐crystal X‐ray diffraction (CCDC number: 2420538), see Figure 2 and S14 of the SI. **10‐mNH_2_** is isostructural to its parent **1‐NH_2_** ^**4+**^ and crystallizes in the cubic space group *Fm*3̅*m*.^[24]^ The unit cell of **10‐mNH_2_** [*a*=42.6196(9) Å and *V*=77416(5) Å^3^] is composed by eight tetrahedral cages built around a central octahedral cavity. Each tetrahedral cage is composed of four [(*n*‐butylCpZr)_3_(OH)_3_O] Zr clusters connected to each other by six ligands. The amino functionalization was found disordered due to the high symmetry of the crystalline material.

**Figure 3:**
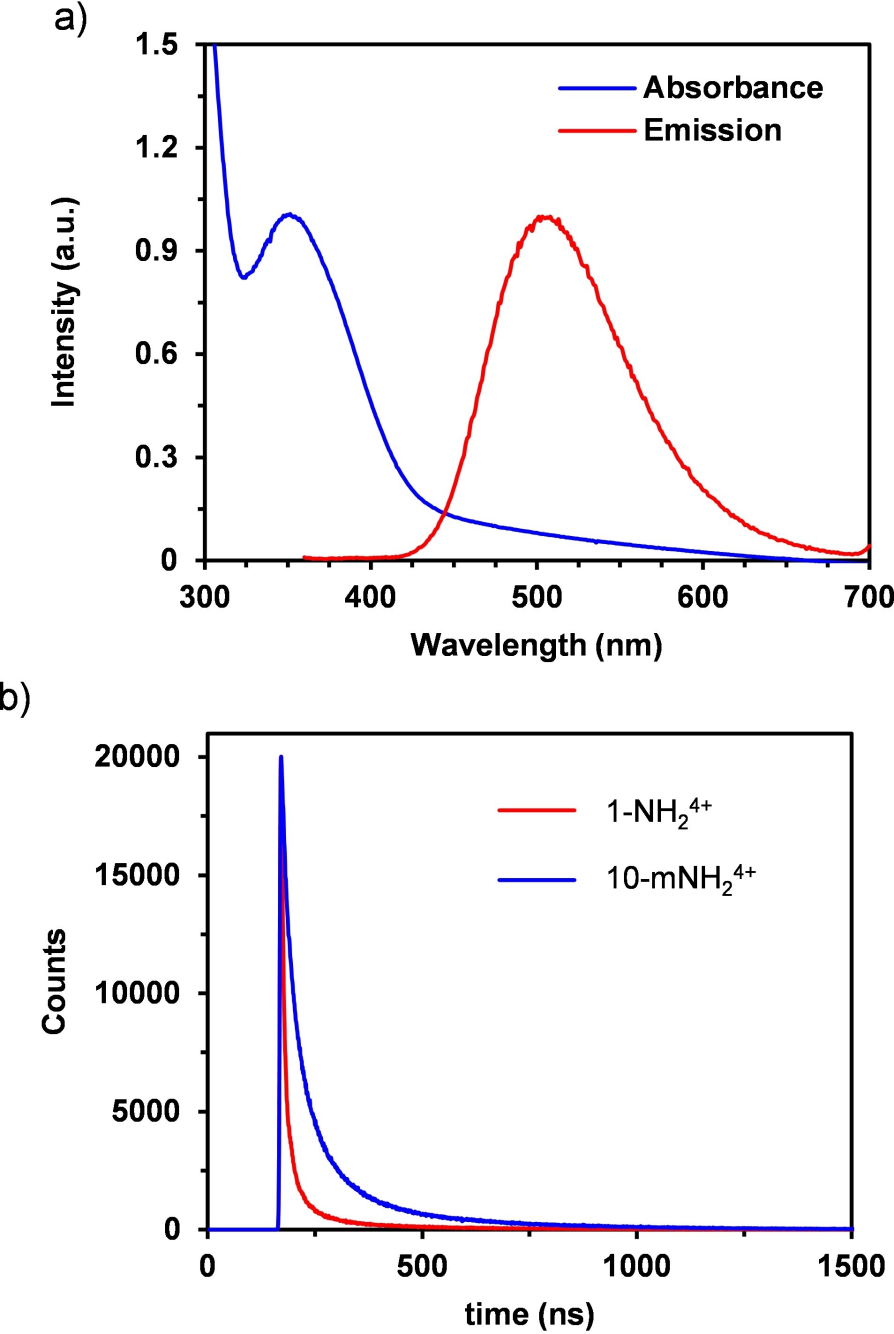
(a) Normalized absorption and emission spectra of a MeOH solution containing 0.2 mM **10‐mNH_2_** ^**4+**^. (b) Excited state decay measured by Time Correlated Single Photon Counting comparing both Zr‐cages explored in this work in a MeOH solution at concentrations of 0.02 mM.

The UV‐vis spectrum of **10‐mNH_2_** ^**4+**^ reveals an absorption band with a maximum centered at 355 nm that is significantly shifted from that corresponding to **1‐NH_2_** ^**4+**^ (390 nm; Figure 3a and S4). In addition, there is an appreciable shoulder at around 400 nm and an absorption tail that extends up to 600 nm likely due to the extended π‐conjugated system of the biphenyl linker. We associate these electronic transitions with a charge transfer from this linker to the Zr_3_(OH)_3_(O) node, as supported by Time Dependent‐Density Functional Theory (TD‐DFT) using a simplified model of the cage limited to one node connected to three linkers. The main transitions relevant to the absorption features have computed wavelengths of 321, 350, and 410 nm (Figure S51–S54). These transitions mainly involve a ligand to metal charge transfer (LMCT) from π‐orbitals centered at the amino‐substituted ring, although with certain participation of the neighbor aromatic ring, to delocalized molecular orbitals with important contributions from the Zr‐based d‐orbitals.

Besides the differences in the UV‐vis spectra between the *orto‐* (**1‐NH_2_** ^**4+**^) and the *meta*‐ (**10‐mNH_2_** ^**4+**^) amino cages, luminescence measurements also reveal a redshift in the emission band from λ_max_=487 nm for **1‐NH_2_** ^**4+**^ to λ_max_=502 nm in the case of the new cage **10‐mNH_2_** ^**4+**^ (Figure 3a). The observed differences in both the emission and absorption spectra result in a higher Stokes shift for **10‐mNH_2_** ^**4+**^ than previously obtained for **1‐NH_2_** ^**4+**^, which could be associated to a higher vibrational relaxation of the corresponding excited state or a better stabilization by solvent reorganization. The higher stabilization of the excited state is also manifested in its lifetime. Time correlated single photon counting experiments of **10‐mNH_2_** ^**4+**^ reveal a biexponential decay akin to **1‐NH_2_** ^**4+**^ but with significantly enhanced lifetimes of 2.7 and 12.5 ns as compared with **1‐NH_2_** ^**4+**^ (1.5 and 8.4 ns) under the same conditions (Figure 3b).^[22]^ Interestingly, switching the solvent to MeCN has an opposite effect on the lifetimes of both cages (Figure S28). In the case of **1‐NH_2_** ^**4+**^, the lifetimes increase up to 3.0 and 12.0 ns, while a significant decrease down to 2.4 and 6.6 ns is obtained for **10‐mNH_2_** ^**4+**^. In contrast, the energy gap between the zeroth vibrational levels of the ground and excited states (E_00_), calculated from the intersection of the normalized emission and absorption spectra, remains 2.8 eV as for **1‐NH_2_** ^**4+**^ (Figure 3a).^[22]^

Overall, these results seem to parallel previous work using **Ti‐BPDC‐o/m‐NH_2_**.^[23]^ Although significant differences between both systems should be acknowledged as evidenced, our observations suggest a more facile charge separation in the case of **1‐NH_2_** ^**4+**^, based on the absorption spectrum, but a greater stabilization of the excited state in the case of **10‐mNH_2_** ^**4+**^ based on the emission spectrum and the Stokes shift. Switching from MeOH to MeCN leads to a decrease in the lifetimes for (**10‐mNH_2_** ^**4+**^)* and an increase for (**1‐NH_2_** ^**4+**^)*. While lower polarity can explain the former case, the increase for (**1‐NH_2_** ^**4+**^)* can be ascribed to intramolecular H‐bonding between the ‐NH_2_ and the carboxylate that reduces the non‐radiative decay pathways involving vibration of these groups, as previously suggested for **Ti‐BPDC‐o‐NH_2_**. This intramolecular H‐bond is more favorable in the case of MeCN due to the lower donor number (14.1 kcal ⋅ mol^−1^) as compared with MeOH (19 kcal ⋅ mol^−1^), resulting in lower H‐bonding competition and the increase of the lifetime for **1‐NH_2_** ^**4+**^.^[25]^

These observations indicate that similar factors govern the photophysical properties of both metal‐organic materials regardless of their structural nature (molecular vs extended), metal node (Ti vs Zr), and phase (solid vs solution phase). While these differences can determine absolute properties such as the redox potential, the similar trends observed are probably dominated by the ligands in both systems. In the case of the **Ti‐BPDC‐m‐NH_2_**, solvent encapsulation within the pores was suggested as responsible for the greater stability when the BPDC‐m‐NH_2_ ligand was used.^[23]^ However, this phenomenon is irrelevant in the case of **10‐mNH_2_** ^**4+**^ due to its discrete, molecular nature, exposing the ‐NH_2_ groups to the bulk solution. We associate the higher stabilization of (**10‐mNH_2_** ^**4+**^)* versus (**1‐NH_2_** ^**4+**^)* to the more extended π‐system of the BPDC‐m‐NH_2_ linker that allows for a better accommodation of the oxidative equivalent (or *hole*). The lower value of the DFT‐computed singlet‐to‐triplet gaps in the case of **10‐mNH_2_** ^**4+**^ (48 kcal⋅mol^−1^) as compared to **1‐NH_2_** ^**4+**^ (55 kcal ⋅ mol^−1^) further supports this idea (Figure S48‐S50).

**Figure 4:**
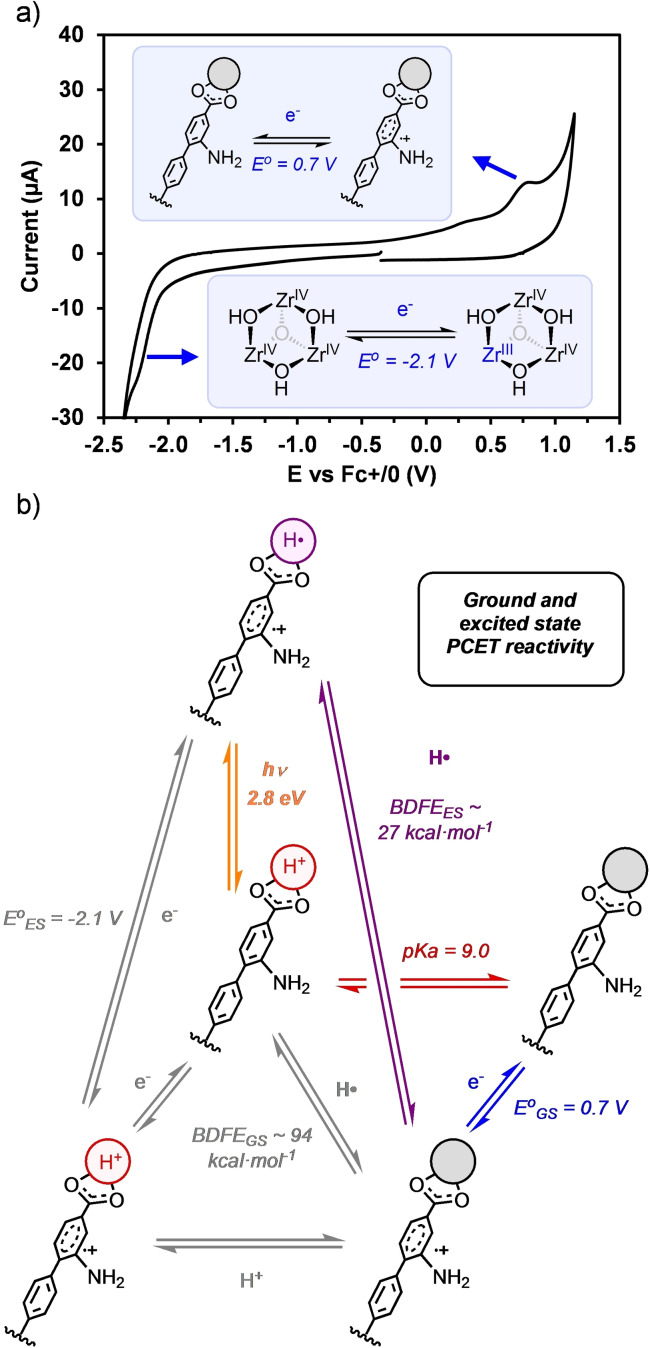
(a) Cyclic voltammetry of a solution containing 1 mM of **10‐xNH_2_** ^**4+**^ in MeOH with [TBA][PF_6_] as the electrolyte, using a glassy carbon working electrode at a scan rate of 100 mV⋅s^−1^. We represent reduction of the node by formation of a Zr(III) ion although DFT suggest a more reasonable charge distribution over the Zr_3_(OH)_3_(O) node. (b) Cubic scheme displaying the ground and excited state PCET reactivity of **10‐xNH_2_** ^**4+**^ based on the thermodynamic and photophysical parameters previously calculated.

The electrochemical behavior of **10‐mNH_2_** ^**4+**^ also exhibits some differences with respect to **1‐NH_2_** ^**4+**^ as inferred from cyclic voltammetry (CV; Figure 4a). While the initial reduction of the Zr nodes in the latter system has a half‐peak potential of around −1.85 V vs Fc^+/0^, **10‐mNH_2_** ^**4+**^ shows a value of −2.1 V vs Fc^+/0^. In addition, this reductive feature appears as a single reduction wave with double intensity as compared to the two reduction peaks identified for **1‐NH_2_** ^**4+**^. These differences are striking given the similar nature of the Zr_3_(OH)_3_(O) node in both cases and can be rationalized in terms of two contributing factors: the cage size and the intramolecular H‐bonding. The cage **10‐mNH_2_** ^**4+**^ has a longer biphenyl linker that provides the nodes with a larger electronic independence, reducing the overall charge density of the molecule. In the case of **1‐NH_2_** ^**4+**^, the positive charge of adjacent nodes has a higher impact on their reduction, likely leading to an overall lower reduction potential and deconvolution of the reduction processes. In addition, the intramolecular H‐bond in **1‐NH_2_** ^**4+**^ can further stabilize the nodes upon one‐electron reduction.

On the other hand, the anodic scan shows an oxidation at a half‐peak potential of 0.70 V vs Fc^+/0^ in the case of **10‐mNH_2_** ^**4+**^ (Figure 4a), cathodically shifted by 250 mV with respect to **1‐NH_2_** ^**4+**^. This redox feature compares well with the CV of the linker (Figure S9) and is consistent with the ligand‐centered oxidation and with the demonstrated decrease of the redox potentials in polyaromatic molecules upon extension of the π‐systems.^[26]^ In addition, this result clearly showcases the better capacity of this ligand to accommodate oxidative equivalents, key in the stabilization of the excited state.

Encouraged by the favorable photophysical and redox properties of **10‐mNH_2_** ^**4+**^, we explored how they could impact the PCET reactivity relative to **1‐NH_2_** ^**4+**^ (Figure 4b). Based on the ground state oxidation potential of the **10‐mNH_2_** ^**4+**^ cage (0.70 V vs Fc^+/0^) and the energy gap between the zeroth vibrational levels of the ground and excited states (E_00_=2.8 eV), the calculated excited state reduction potential for (**10‐mNH_2_** ^**4+**^)* is −2.1 V vs Fc^+/0^ according to Eq. 1. This value reflects the more reducing character of this new cage by 250 mV, as previously demonstrated also in the ground state, which translates into a lower bond dissociation free energy (BDFE) of 27 kcal ⋅ mol^−1^ calculated using the Bordwell equation (Eq. 2). For the latter, the p*K*_a_ is calculated to be 9.0 by UV‐vis titration resulting in a similar value as for **1‐NH_2_** ^**4+**^ (Figure S6–S8). This is associated with the relatively acidic hydroxo‐bridges in the Zr_3_(OH)_3_(O) nodes, that are similar in both cages, acting as the proton source.
(1)

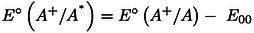

(2) $$BDFE=1.37\cdot {pK}_{a}+23.06\cdot E^{o}+C_{G}$$

The longer lifetime and lower BDFE of (**10‐mNH_2_** ^**4+**^)* suggest an enhanced ES PCET compared to **1‐NH_2_** ^**4+**^. We thus evaluated the comparative reactivity of this new cage towards photocatalytic reduction of acetophenone as a model substrate, which forms a ketyl radical intermediate with a BDFE_O−H_=38 kcal ⋅ mol^−1^ (Table 1).^[22]^ Irradiation with 440 nm LED of an isopropanol solution containing **10‐mNH_2_** ^**4+**^ (1 mM) and acetophenone (50 equiv.) resulted in the formation of 70 % of the pinacol product 3‐diphenylbutane‐2,3‐diol (Table 1‐ entry 2). This product forms via an initial PCET from (**10‐mNH_2_** ^**4+**^)* to acetophenone followed by diffusion limited coupling of the resulting ketyl radical. Excitingly, the yield of this reaction is 40 % higher than in the case of **1‐NH_2_** ^**4+**^ (Table 1‐ entry 1). This difference becomes even higher at lower substrate loading (25 equiv.) with almost full conversion when using **10‐mNH_2_** ^**4+**^ versus the 37 % achieved with **1‐NH_2_** ^**4+**^.

**Table 1:** Results from the photocatalytic reduction of acetophenone by the Zr‐cages explored in this work under different conditions.

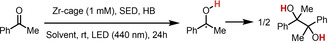				
Entry	Zr‐cage	[Acetophenone]	Solvent	Yield
1	**1‐NH_2_** ^**4+**^	50 mM	*i‐*PrOH	25 %
2	**10‐mNH_2_** ^**4+**^	50 mM	*i‐*PrOH	70 %
3	**1‐NH_2_** ^**4+**^	25 mM	*i‐*PrOH	37 %
4	**10‐mNH_2_** ^**4+**^	25 mM	*i‐*PrOH	99 %
5	**1‐NH_2_** ^**4+**^	50 mM	MeCN	86 %^a^
6	**10‐mNH_2_** ^**4+**^	50 mM	MeCN	97 %
7	**1‐NH_2_** ^**4+**^	50 mM	MeCN^b^	80 %
8	**10‐mNH_2_** ^**4+**^	50 mM	MeCN^b^	18 %

^a^ From reference 22. ^b^With TEA/TEAH+ buffer added. **Notes**: SED is sacrificial electron donor and HB is the proton donor. In the case of iPrOH, the solvent was employed as sacrificial electron and proton donor. In MeCN, the SED was TEA (1 M) and water (3 M) was employed as proton donor.

When this reaction is performed in MeCN under previously optimized conditions, using 1 M triethylamine (TEA) as the sacrificial electron donor (SED) and H_2_O (1.5 M) as the proton source, the yield increases up to 99 % for a 50 equiv. loading of acetophenone. These results show once again the enhanced catalytic performance of **10‐mNH_2_** ^**4+**^, although with a more attenuated difference in MeCN of around 10%. The photocatalytic experiments were also performed in MeCN with TEA/TEAH^+^ as the buffer (pK_a_ 18.8 in MeCN) for a better control of the proton activity. The results show a partial decrease of the yield for **10‐mNH_2_** ^**4+**^ that becomes larger in the case of **1‐NH_2_** ^**4+**^ and could tentatively be associated to the more basic conditions imposed by the buffer (pK_a_ of 18.8) leading to partial deprotonation the Zr‐nodes. Nonetheless, the difference in the photocatalytic performance between both cages remains. (Table 1, entry 7–8).

**Figure 5:**
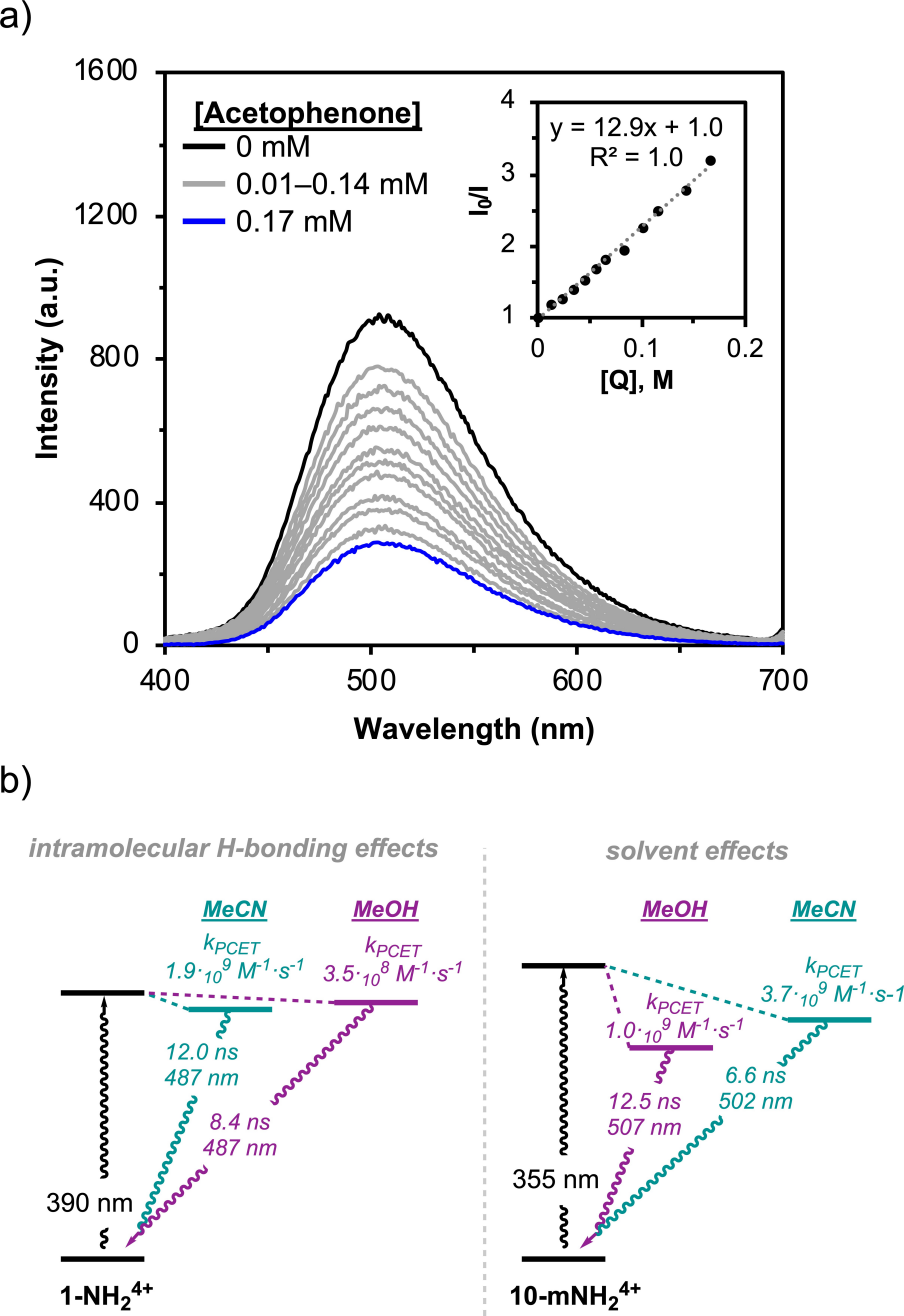
(a) Fluorescence quenching upon addition of acetophenone to a solution of **10‐mNH_2_** ^**4+**^ (0.2 mM) in MeOH. Inset: Stern–Volmer plot for the quenching of 0.2 mM **1‐NH_2_** ^**4+**^ with increasing concentrations of acetophenone in MeOH. (b) Simplified Jablonski diagrams for both cages summarizing the relevant features and the proposed effect of the solvent.

Excited by these results, we interrogated the PCET rates for **10‐mNH_2_** ^**4+**^ based on photoluminescence quenching experiments to explore the kinetic grounds for the difference in reactivity (Figure 5a). The addition of acetophenone to a MeOH solution of **10‐mNH_2_** ^**4+**^ (0.2 mM) resulted in a significant decrease of the emission intensity. The quenching data was fitted according to the Stern‐Volmer equation (Eq. 3) revealing a linear correlation between I_0_/I and [acetophenone] (Figure 5a). In this equation, I_0_ and I are the emission intensity in the absence and presence of acetophenone respectively, K_SV_ is the Stern‐Volmer constant, [Q] is the concentration of the quencher (acetophenone), and τ_0_ is the lifetime. We extracted K_SV_ from the corresponding linear fit, which allowed to further calculate the rate constant for the ES PCET step (k_PCET_) considering the lifetime of **10‐mNH_2_** ^**4+**^. The latter resulted in a k_PCET_=1.0 ⋅ 10^9^ M^−1^ ⋅ s^−1^ that is 3 times higher than that previously calculated for **1‐NH_2_** ^**4+**^ (3.5 ⋅ 10^8^ M^−1^ ⋅ s^−1^).^[22]^ Thus, the more pronounced quenching in the case of **10‐mNH_2_** ^**4+**^ is a combination of the longer excited‐state lifetime and the intrinsically faster PCET step consistent with the lower BDFE. In addition, these kinetic differences are also reflected in the quantum yield of the photochemical PCET reaction, Φ. We find a Φ=0.04 that, despite remaining modest, duplicates the value previously obtained for **1‐NH_2_** ^**4+**^.

(3) $$\frac{I_{0}}{I}=1+K_{SV}\cdot \left[Q\right]=1+k_{PCET}\cdot \tau_{0}\cdot \left[Acetophenone\right]$$

Performing the quenching experiments in MeCN for both **1‐NH_2_** ^**4+**^ and **10‐mNH_2_** ^**4+**^ shows a more attenuated difference in the K_SV_ of only 4 M^−1^, consistent with the reduced difference found in the pinacol yields from the photocatalytic reactions (Figure S31 and S32). In this case, MeCN induces a decrease of the lifetime for **10‐mNH_2_** ^**4+**^ but an increase for **1‐NH_2_** ^**4+**^, contributing to reducing the reactivity gap between both systems. Despite that opposite effect, we found a significant enhancement of the k_PCET_ using both **1‐NH_2_** ^**4+**^ and **10‐mNH_2_** ^**4+**^ to 1.9 ⋅ 10^9^ M^−1^ ⋅ s^−1^ and 3.9 ⋅ 10^9^ M^−1^ ⋅ s^−1^ respectively. The latter overcompensates the lifetime changes, resulting again in an overall kinetic advantage for **10‐mNH_2_** ^**4+**^. The similar enhancement in the PCET rate exerted by MeCN is interesting and we hypothesize that it might be related to the lower reorganization energy upon PCET in this solvent. As aforementioned, the higher donor number of MeOH most likely favors H‐bonding interactions with the node and/or acetophenone that somewhat hamper the proton and electron transfer. The effect of the different dielectric constants is expected to be rather small, especially considering this redox neutral reaction.[^27^, ^28^] These conclusions have been summarized in Figure 5b.

These findings can be analyzed in the context of those obtained using **Ti‐BPDC‐o/m‐NH_2_** as H_2_ evolution photocatalysts, although a cautionary comparison must be made due to the different nature of the metal nodes.^[23]^ While the *orto*‐ position of the ‐NH_2_ group with respect to the carboxylate enhances the H_2_ evolution using **BPDC‐o‐NH_2_** in Ti‐MOFs, it proves negative for the PCET reactivity of related Zr‐cages due to its role in decreasing the reduction power of the node. This difference in reactivity can be partially compensated by solvent selection to promote intramolecular H‐bonding with the node that extends the excited state lifetime. However, the *meta*‐ position of the ‐NH_2_ leads to an overall more efficient ES PCET reaction, resulting in higher kinetics and quantum/chemical yields.

## Conclusions

The development of molecular mediators that utilize light to promote PCET is a promising tool for solar‐to‐chemical technologies. Building on our previous results using a molecular Zr‐cage as photocatalytic PCET mediator, we report here the design of a new platform with a larger cage size and a *meta‐* position of the ‐NH_2_ group relative to the carboxylate. These structural aspects have a key impact on the photophysical and redox properties of the cage, increasing the stability of the excited state and decreasing its reduction potential and thus, the BDFE. These features, which are typically modulated by electronic tuning via inductive effects, determine a more efficient photocatalytic PCET reaction illustrated by the higher chemical and quantum yields and the faster rate constants for the reduction of acetophenone. These findings shine light on the factors that have an impact on the excited state PCET reactivity and manifest the potential of underexploited tools to control the reactivity such as cage size and intramolecular H‐bonding.

## Supporting Information

The authors have cited additional references within the Supporting Information.[^22^, ^24^, ^29^, ^30^, ^31^, ^32^, ^33^, ^34^, ^35^, ^36^, ^37^, ^38^, ^39^] CCDC 2420538 contains the supplementary crystallographic datum for this paper. This datum can be obtained free of charge from The Cambridge Crystallographic Data Centre via http://www.ccdc.cam.ac.uk/

## Conflict of Interests

The authors declare no conflict of interest.

1

## Supporting information

As a service to our authors and readers, this journal provides supporting information supplied by the authors. Such materials are peer reviewed and may be re‐organized for online delivery, but are not copy‐edited or typeset. Technical support issues arising from supporting information (other than missing files) should be addressed to the authors.

Supporting Information

## Data Availability

The data that support the findings of this study are available in the supplementary material of this article.
