# Supplementary material for: Enhanced Photocatalytic Proton‐Coupled Electron Transfer by Ligand Design in a Zr Coordination Cage
Source: ChemSusChem. 2025 Mar 19;18(11):e202500219. doi: 10.1002/cssc.202500219 (PMC12131689; doi:10.1002/cssc.202500219)
Supplement: Supplementary file 1 — Supporting Information [file CSSC-18-e202500219-s001.pdf]

ChemSusChem

Supporting Information

Enhanced Photocatalytic Proton-Coupled Electron Transfer by Ligand Design in a Zr Coordination Cage

Pedro J. Jabalera-Ortiz, Alvaro M. Rodriguez-Jimenez, Rebecca Vismara, Pedro Delgado, Natalia M. Padial, Jorge A. R. Navarro, and Pablo Garrido-Barros*

Supporting Information for

Enhanced Photocatalytic Proton-Coupled Electron Transfer by Ligand Design in a Zr Coordination Cage

Pedro J. Jabalera-Ortiz, Alvaro M. Rodriguez-Jimenez, Rebecca Vismara, Pedro Delgado, Natalia M. Padial, Jorge A. R. Navarro, and Pablo Garrido-Barros*

Departamento de Química Inorgánica, Facultad de Ciencias, Universidad de Granada and Unidad de Excelencia en Química (UEQ), Avda. Fuente Nueva s/n, 18071, Granada, Spain. E-mail: pgarridobarros@ugr.es

Table of contents

S.1. General considerations. 3

S.1.1. Materials and reagents. 3

S.1.2. Physical and chemical methods of characterization. 3

S.1.3. Computational details 4

S.2. Synthesis of materials. 5

S.2.1. Elemental analysis 6

S.2.2. TOF-MS ES+ 7

S.3. Physical and chemical characterization 8

S.3.1. Fourier Transform Infrared Spectroscopy (FTIR). 8

S.3.2. UV-vis spectroscopy 9

S.3.3. Electrochemistry 12

S.3.4. Fluorescence emission 21

S.3.5. Crystal data 22

S.4. Lifetime measurements 26

S.5. Stern-Volmer 28

S.6. Quantum yield of the photochemical PCET reaction 31

S.7. Photocatalytic reactions 33

S.8. DFT calculations 42

S9. References 62

S.1. General considerations.

S.1.1. Materials and reagents.

Chemical reagents and solvents were purchased at commercial sources and used without additional purification. Dried solvents were purchased from Sigma Aldrich and sparged with N_2 before their use. Deuterated DMSO-d_6, MeOD and $CDCl_3$ solvents (D, 99.9% with a purity of 99.5%) were purchased from Cambridge Isotope Laboratories, Inc., and used as received. Teflon-coated magnetic stir bars were soaked in concentrated nitric acid for at least 1 h, washed repeatedly with deionized water then acetone, and dried in an oven prior to use.

S.1.2. Physical and chemical methods of characterization.

^{1}H Nuclear Magnetic Resonance Spectroscopy (RMN) data were recorded on a 400 MHz BRUKER Nanobay Avance III HD High-Definition spectrometer and the spectra were internally referenced to solvent signals

UV-vis spectroscopy was performed in a SHIMADZU UV-1800 UV/VIS Scanning Spectrophotometer.

Cyclic voltammetry was carried out on a WaveNow Wireless Potentiostat/Galvanostat, using a one-compartment three-electrode cell, a glassy carbon (GC) disk as the working electrode, a Pt disk as the counter electrode, and an Ag/AgOTf (5 mM) reference electrode. Details for the CVs are noted as they appear. $E_{1/2}$ values for the reversible waves were obtained from the half potential between the oxidative and reductive peaks. All the reported potentials are referenced to the ferrocenium/ferrocene couple ($Fc^{+/0}$), which has been used as an internal standard. The GC disk electrode for cyclic voltammetry was polished using 1, 0.3 and 0.05 µm alumina powder.

Steady-state luminescence was recorded on a Cary Eclipse Fluorescence Spectrometer. Excitation was provided by a 450 W Xe arc lamp, wavelength-selected with a 0.25 m monochromator. Luminescence was collected at 90° with reflective optics. All spectra were corrected for instrument response.

Elemental analysis was carried out on a THERMO SCIENTIFIC Model Flash 2000 elemental analyzer.

TOF-MS ES+ measurements were recorded in a WATERS model LCT Premier XE mass spectrometer.

S.1.3. Computational details

All DFT calculations were performed in the Gaussian 09,[1] using the TPSS (meta-GGA)[2] functional. The more appropriate basis set for heavier atoms such as Zr, def2-TZV[3,4] was employed for TD-DFT calculations on all atoms. Due to the high computational cost of this basis set, the simpler def2-SVP[3,4] was used on all atoms for DFT geometry optimizations. Solvent effects were simulated using the SMD[5] implicit solvation modelling with methanol, allowing for direct comparison with experimentally available data.

All calculations were computed in solution without symmetry restrictions. All calculated structures were stationary points as confirmed by single-point vibrational frequency calculations. To reproduce the absorption spectra of the studied molecules, 50 electronic states were calculated using TD-DFT. Free energy corrections were calculated at 298.15 K and 105 Pa pressure, including zero-point energy corrections (ZPE). Unless otherwise mentioned, all reported energy values are free energies in solution under standard state conditions. The TD-DFT and DFT calculations with the optimized xyz coordinates are available in the ioChem-BD database.[6]

S.2. Synthesis of materials.

The synthesis of $[((^{nBu}CpZr)_3(OH)_3O)_4(2\text{-aminoterephtalate})_6]Cl_4$ ($1\text{-}NH_2^{4+}$) was carried out according to the procedure stablished by Delgado *et al.* (2022)[7] and physical and chemical characterization of the resulting material was done according to Jabalera-Ortiz *et al.* (2024)[8]. The synthesis of the organic acids employed here was done by adding excess HCl to the corresponding base in Et_2O, followed by filtration and successive washing with Et_2O.

The synthesis of $[((^{nBu}CpZr)_3(OH)_3O)_4$(2-amino-[1,1'-biphenyl]-4,4'-dicarboxilate)$_6]Cl_4\cdot 2HCl$ (**10-mNH$_2^{4+}$**) was done as follows. In a 5 mL vial, 20 mg of 2-amino-[1,1'-biphenyl]-4,4'-dicarboxilic acid and 55.5 of dibutylzirconocene dichloride were dissolve in 450 µL of DMF and 220 µL of carbon tetrachloride. Then, 220 µL of H_2O was added drop by drop and the mixture was heated 12 h at 60 ºC. The solid powder was recovered by washing with DMF (3x5 mL) and hexane (1x5 mL) and dried at room temperature. Yield: 30 mg, 40%. ^1H NMR (400.1 MHz, CD_3OD): δ (ppm) 8.00 (s, CHO, DMF), 7.40 (m, 42 H, Ph), 6.56 (t, J = 4 Hz, 24 H, Cp), 6.40 (t, J = 4 Hz, 24 H, Cp), 3.01 (s, CH_3, DMF), 2.88 (s, CH_3, DMF), 1.65 (q, J = 8 Hz, 24 H, CH_2, n-butyl), 1.35 (m, 24 H, CH_2, n-butyl), 0.91 (t, J = 8 Hz, 36 H, CH_3, n-butyl).

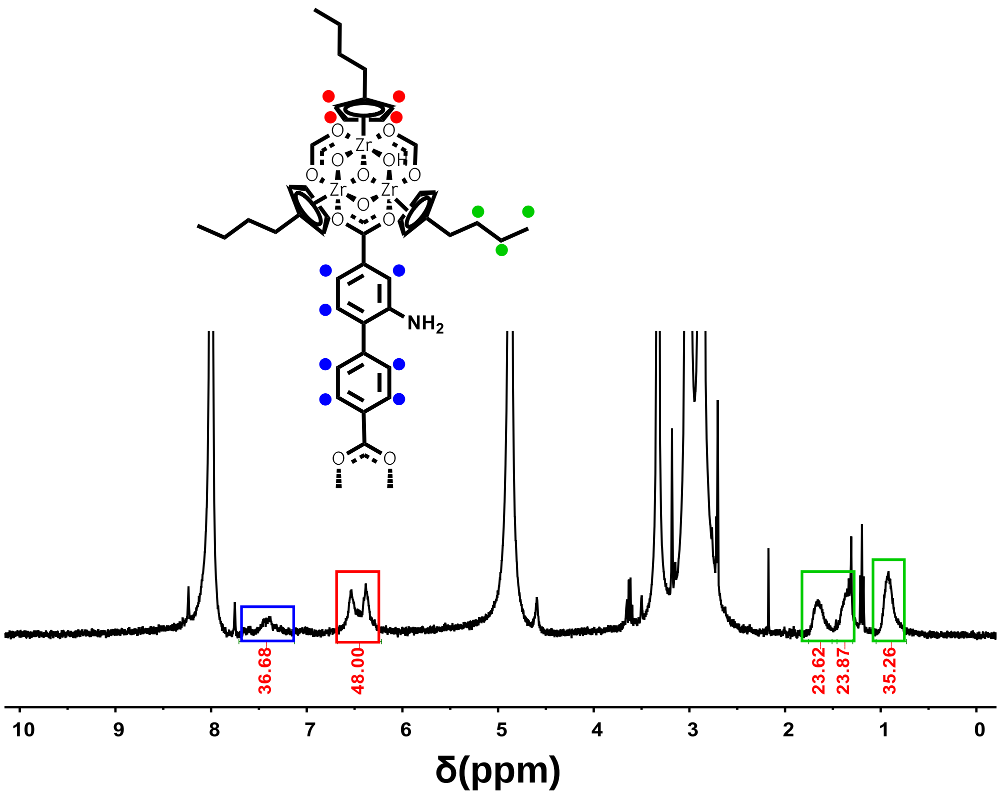

Figure S1. ^{1}H NMR spectra of **10-mNH$_2$$^{4+}$** in MeOD.

S.2.1. Elemental analysis

Prior to measure, 10 mg of **10-mNH$_2$$^{4+}$** were put at 60 ºC and under vacuum during 24 h in order to eliminate as much solvent as possible.

Table S1. Elemental analysis of **10-mNH$_2$$^{4+}$**.

**Sample**	**Elements %**		
	**N**	**C**	**H**
**10-NH$_2$$^{4+}$**	2.20	49.34	5.12
**Simulated (With 11 H$_2$O and 1 DMF)**	2.08	49.96	5.41
**Simulated**	1.9	52.10	5.06

S.2.2. TOF-MS ES+

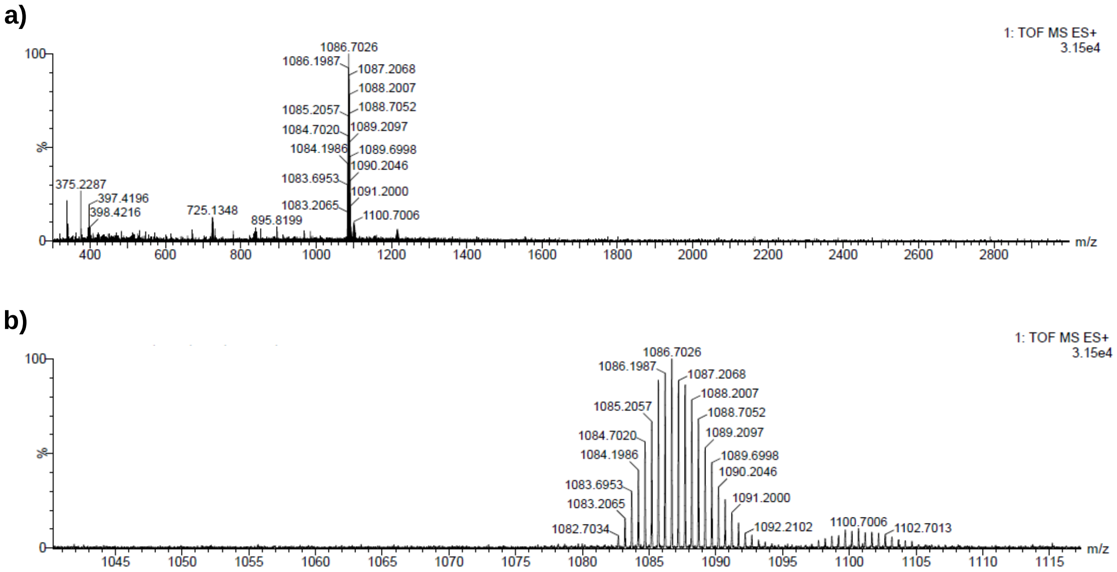

Figure S2. MALDI-TOF-MS measurement of **10-mNH$_2^{4+}$** showed a m/z peak of 1086.7026 corresponding to the [M/4] of the cage.

S.3. Physical and chemical characterization

S.3.1. Fourier Transform Infrared Spectroscopy (FTIR).

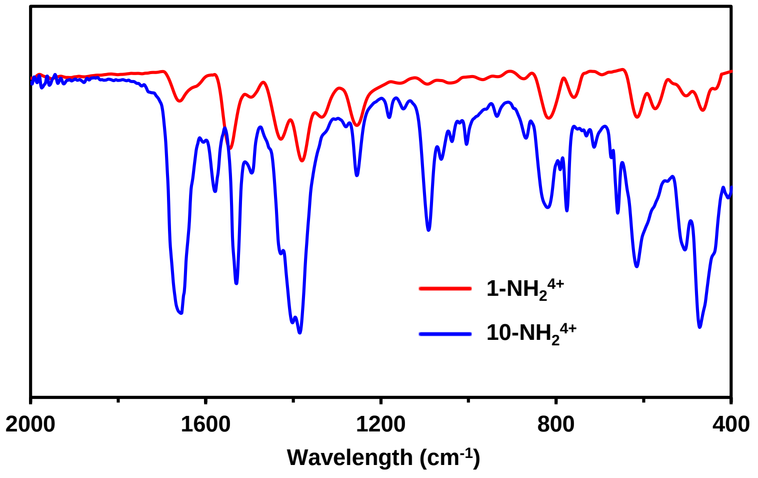

Figure S3. FTIR spectra of **1-NH$_2$$^{4+}$** and **10-mNH$_2$$^{4+}$**.

S.3.2. UV-vis spectroscopy

Solutions with 10^{-2} mM of **1-NH$_2$$^{4+}$**, **10-mNH$_2$$^{4+}$** or 2-amino-[1,1'-biphenyl]-4,4'-dicarboxilic acid were prepared in dried methanol.

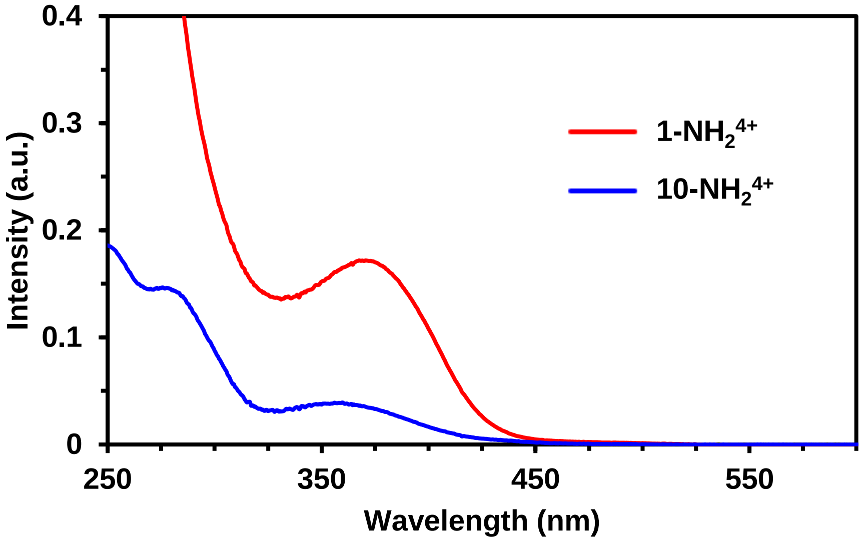

Figure S4. UV-vis spectra of **1-NH$_2$$^{4+}$** and **10-mNH$_2$$^{4+}$** in methanol.

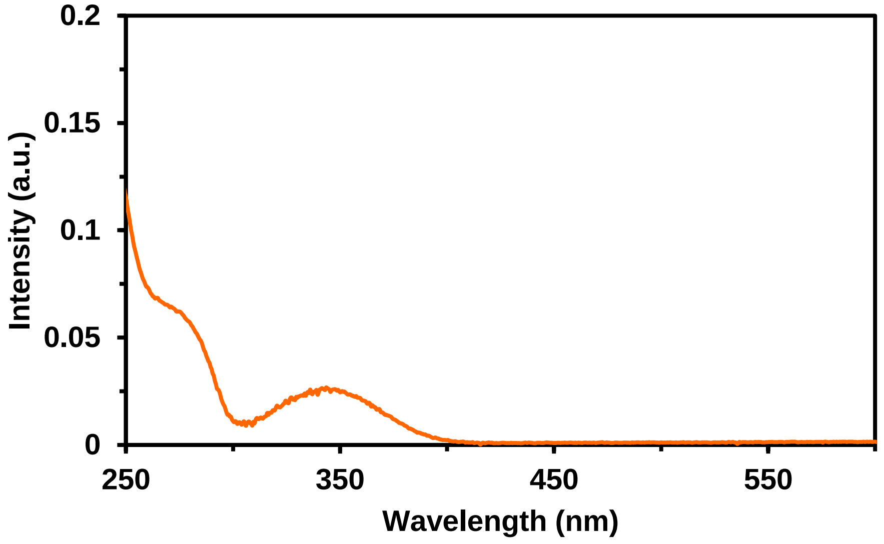

Figure S5. UV-vis spectra of 2-amino-[1,1'-biphenyl]-4,4'-dicarboxilic acid in methanol.

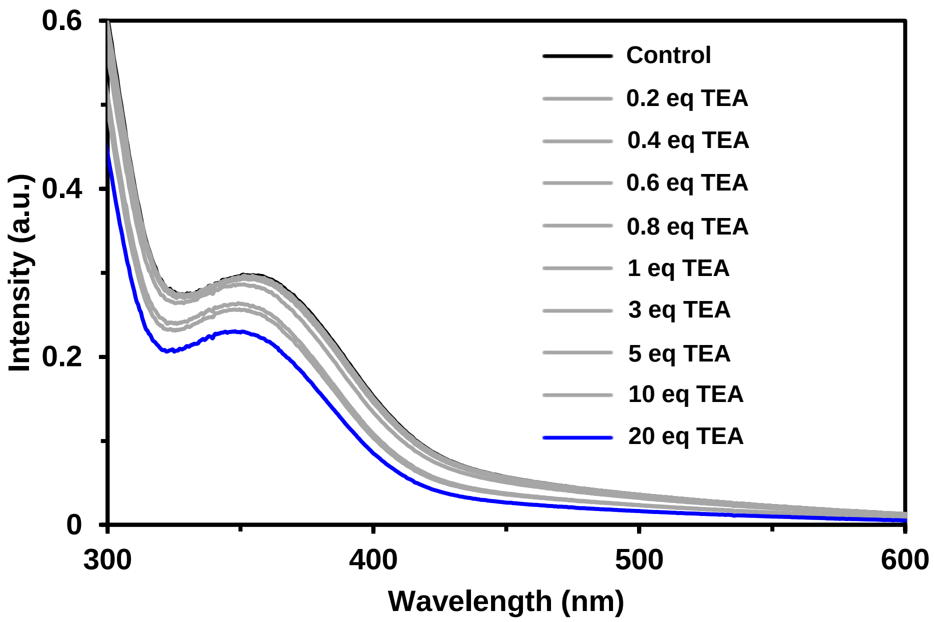

Figure S6. UV-vis spectra of **10-mNH$_2$$^{4+}$** in methanol with addition of subsequent equivalents of TEA.

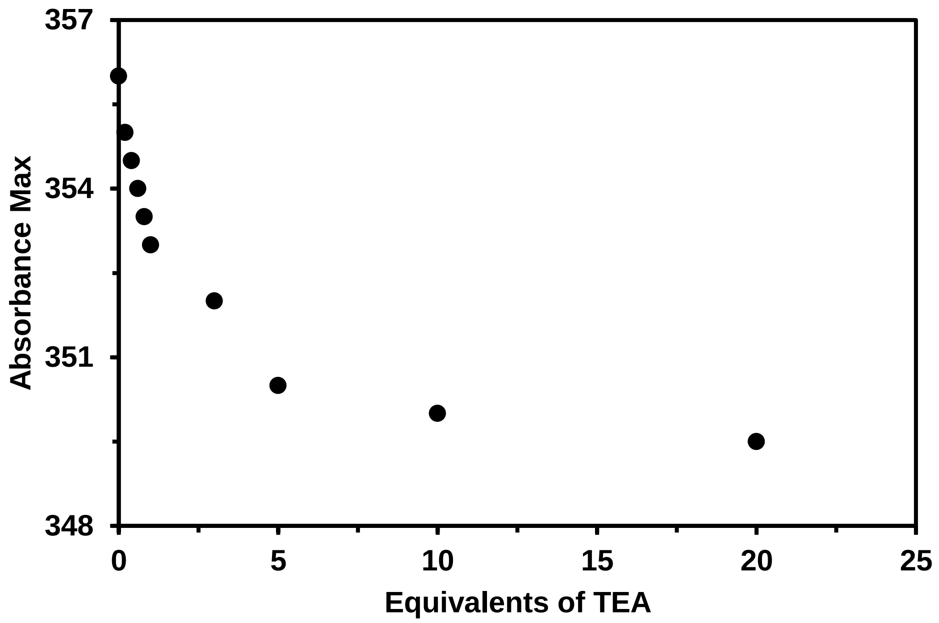

Figure S7. Plot of the maximum absorbance of **10-mNH$_2$$^{4+}$** versus the added equivalents of TEA.

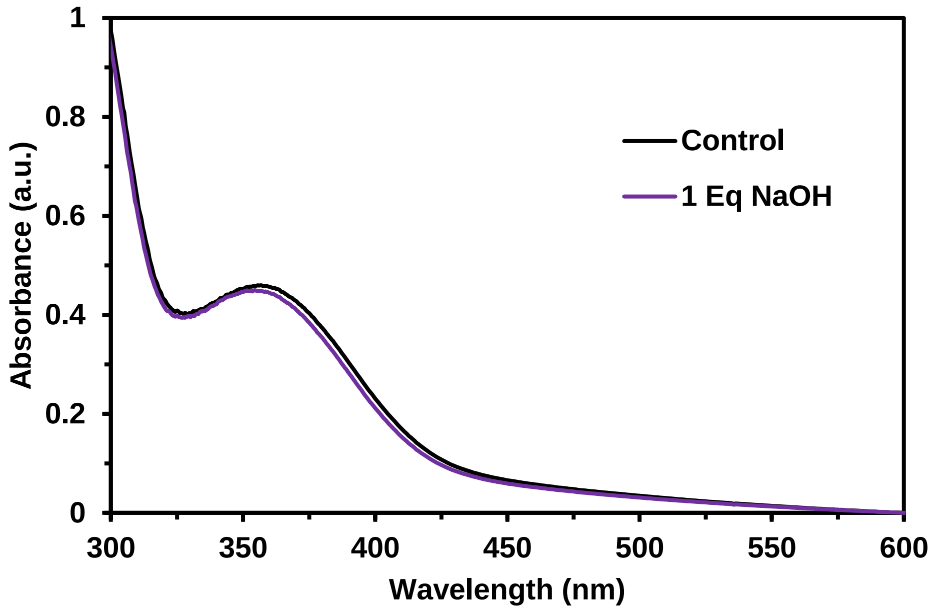

Figure S8. UV-vis spectra of **10-mNH$_2$$^{4+}$** in methanol with addition of one equivalent of NaOH.

S.3.3. Electrochemistry

In a 26 mL glass vial, 4 mg of 1-NH_2^{4+} (0.033M), 4.5 mg of 10-mNH_2^{4+} (0.033M) or 0.25 mg of 2-amino-[1,1'-biphenyl]-4,4'-dicarboxilic acid (0.033M) and 194 mg of tetrabutylammonium hexafluorophosphate ($TBAPF_6$) (0.1 M) were dissolved in 5 mL of dried MeOH or MeCN. The solution was then put under a constant flow of argon (Ar) during 15 min before measurement to eliminate the presence of O_2.

The redox potentials were extracted from the CV using the half-peak potential due to the irreversible character of the waves following the literature discussion.[9] While this still might lead to certain error in their determination, the comparative analysis between 1-NH_2^{4+} and 10-NH_2^{4+} minimizes its impact

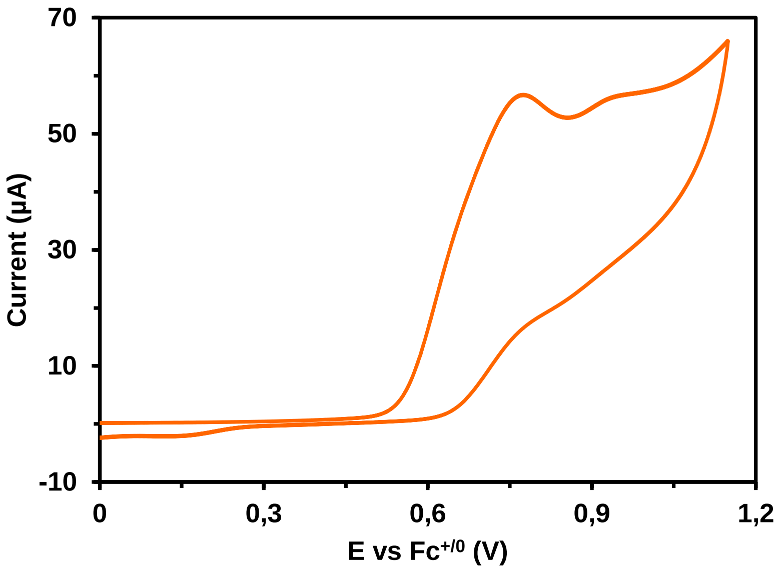

Figure S9. Cyclic voltammetry of 2-amino-[1,1'-biphenyl]-4,4'-dicarboxilic acid in methanol, showing the oxidation waves.

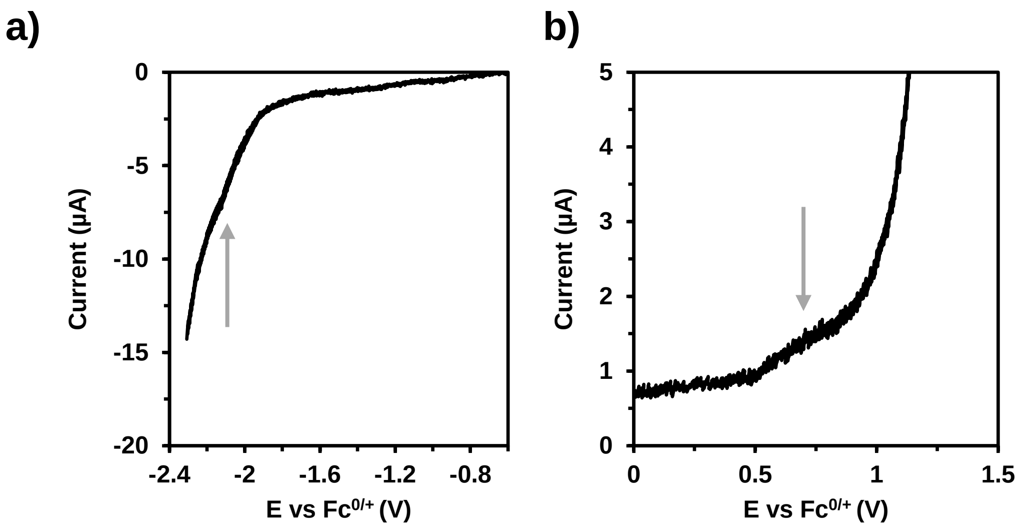

Figure S10. Differential pulse voltammetry of **10-mNH$_2$$^{4+}$** in methanol, showing the reduction (a) and oxidation (b) waves.

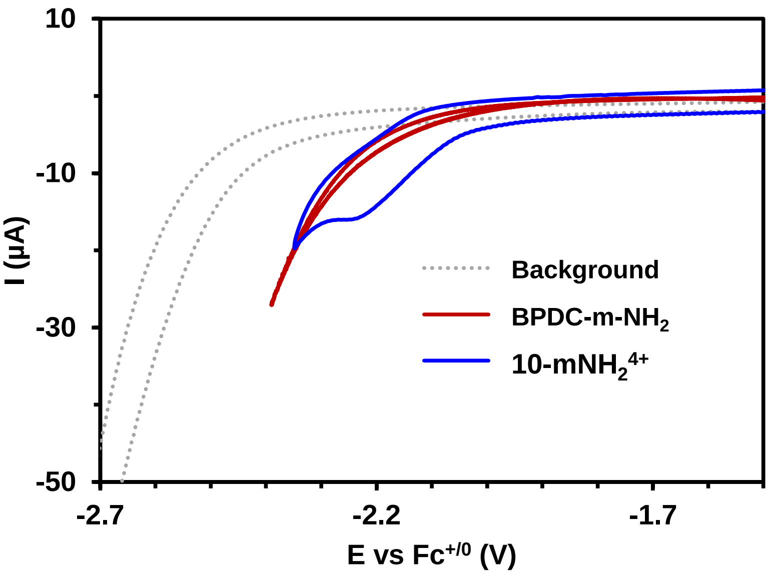

Figure S11. Cyclic voltammetry showing the reduction waves of 2-amino-[1,1'-biphenyl]-4,4'-dicarboxilic acid (red) and **10-mNH$_2$$^{4+}$** (blue) in methanol with TBAPF$_6$ (gray is the background electrolyte).

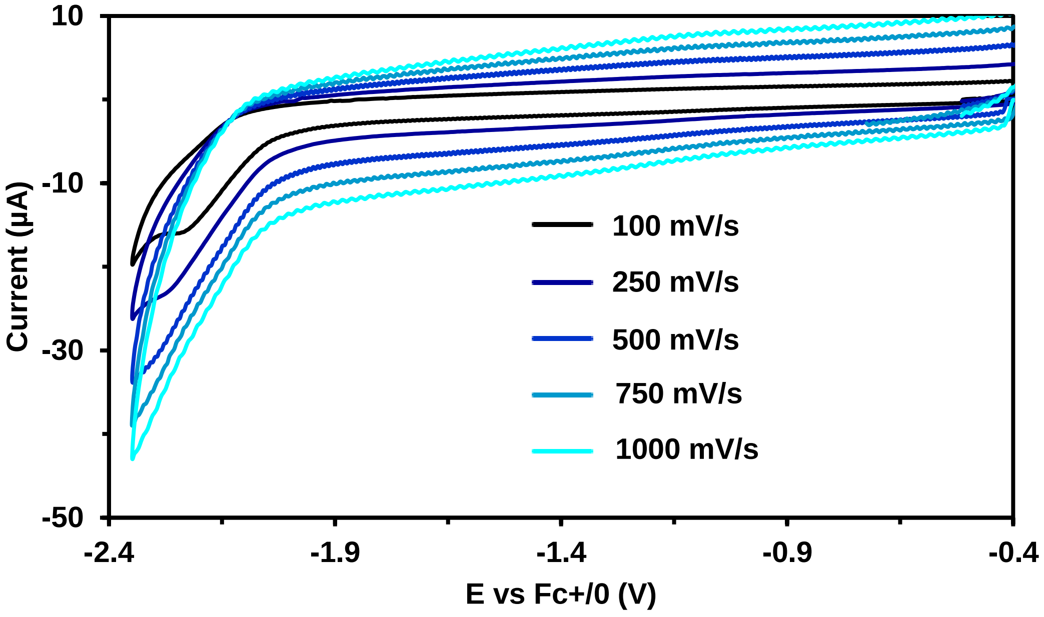

Figure S12. Cyclic voltammetry of **10-mNH$_2$$^{4+}$** in methanol with different scant rates.

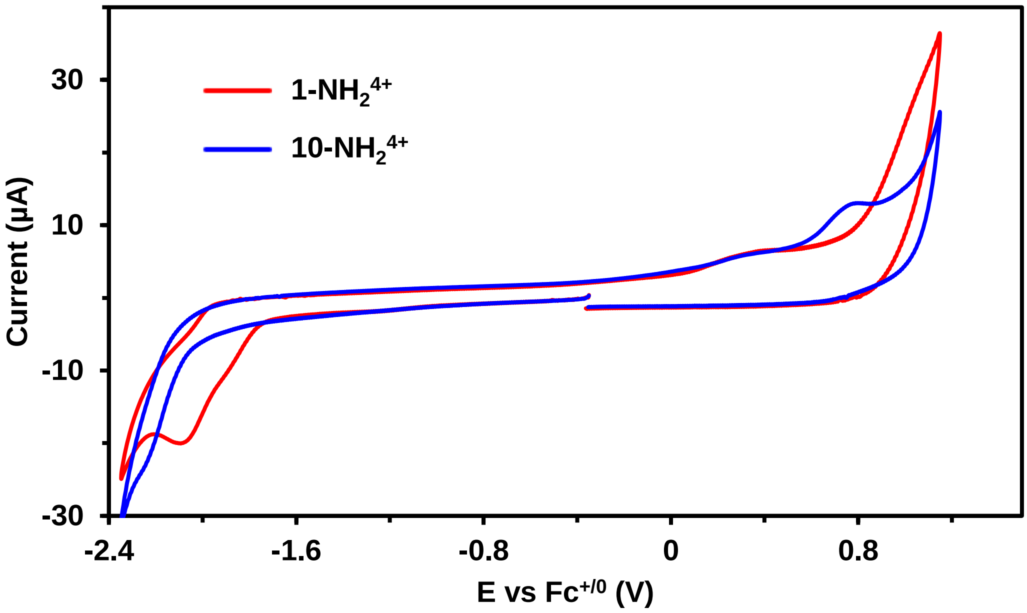

Figure S13. Cyclic voltammetry of **1-NH$_2$$^{4+}$** and **10-mNH$_2$$^{4+}$** in methanol.

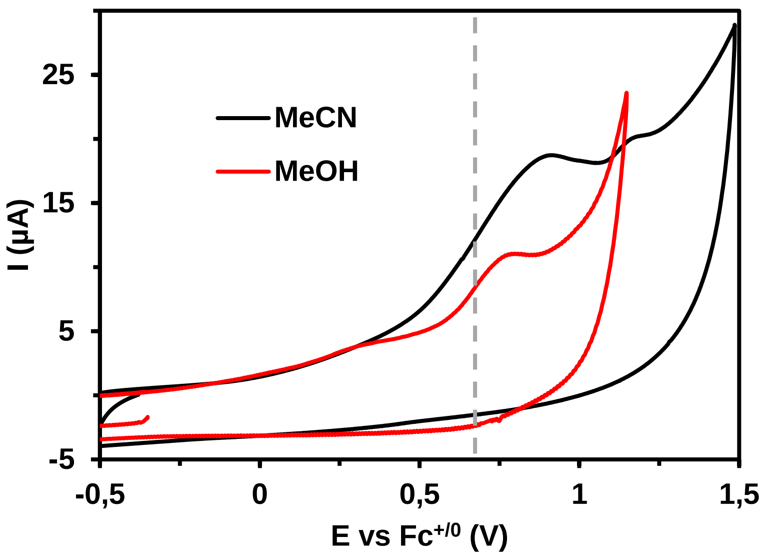

Figure S14. Cyclic voltammetry of **10-mNH$_2$$^{4+}$** in acetonitrile (black) and methanol (red), showing the oxidation waves.

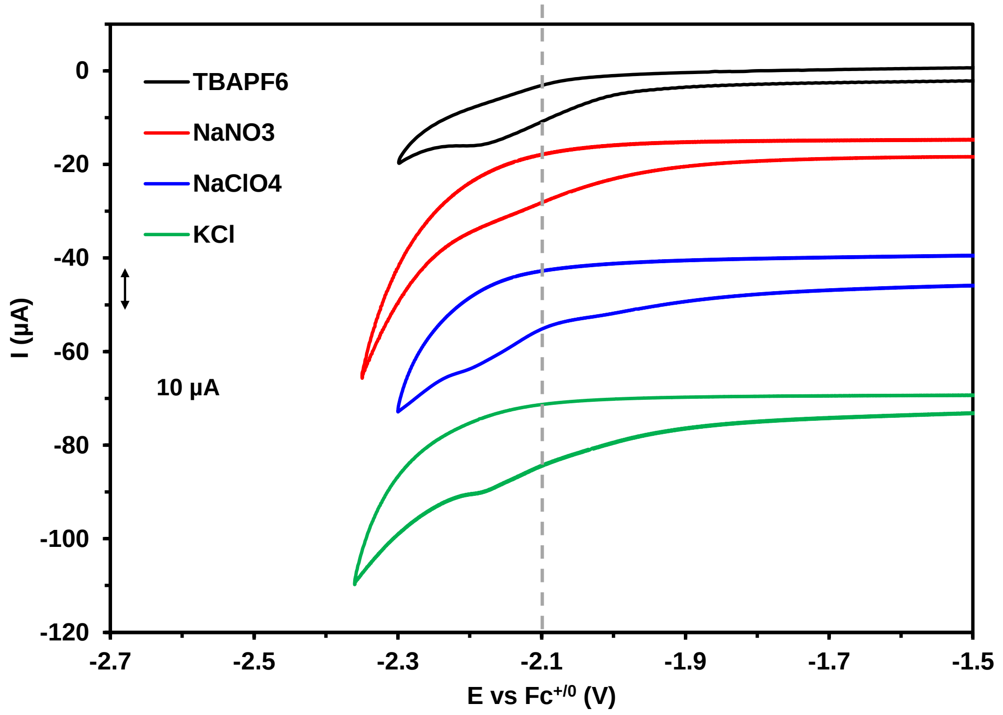

Figure S15. Cyclic voltammetry of **10-mNH$_2$$^{4+}$** in methanol, showing the reduction waves, employing different electrolytes.

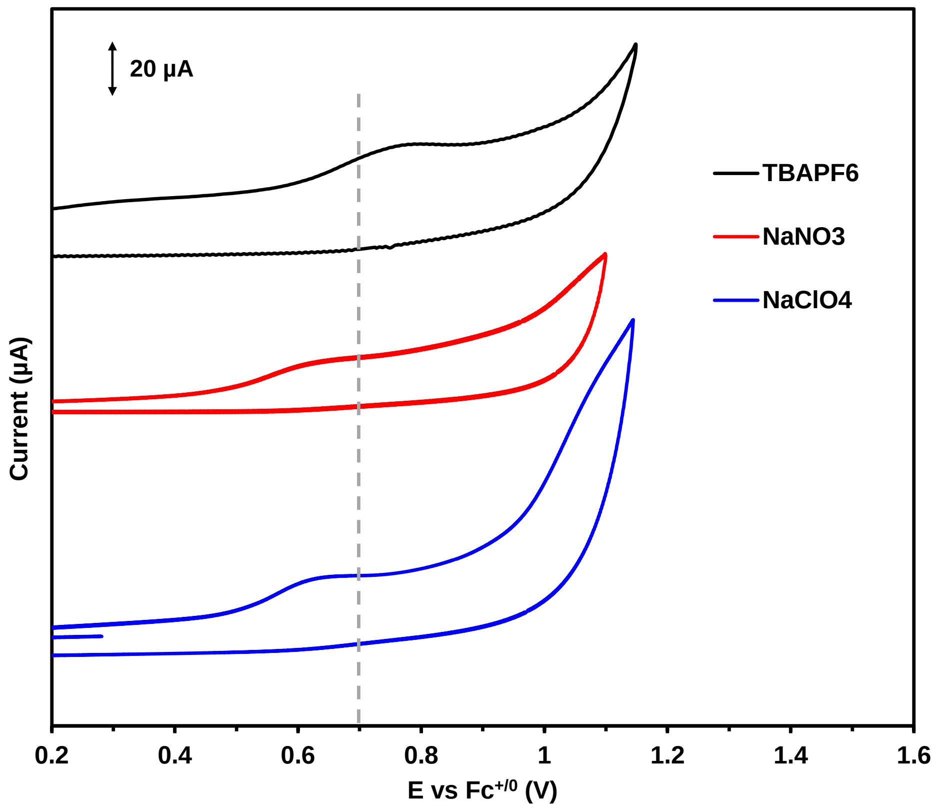

Figure S16. Cyclic voltammetry of **10-mNH$_2$$^{4+}$** in methanol, showing the oxidation waves, employing different electrolytes.

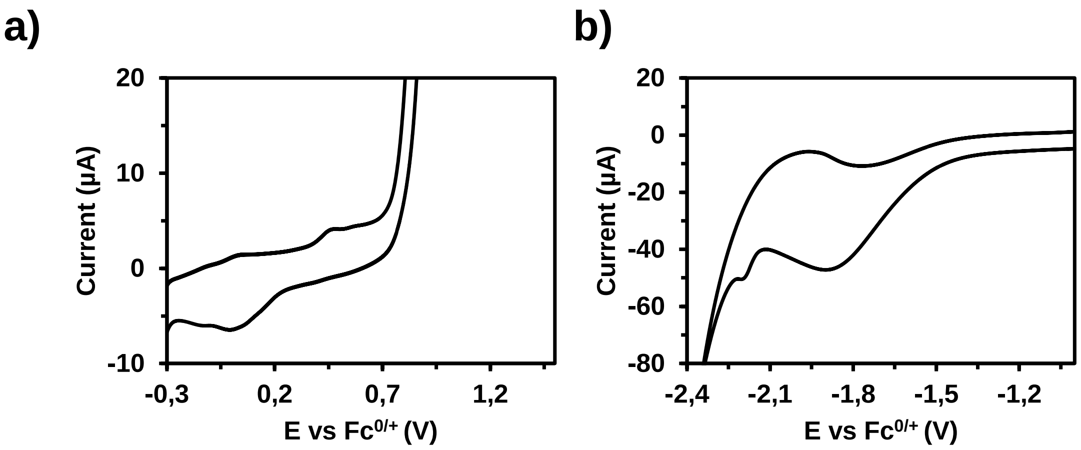

Figure S17. Cyclic voltammetry of **10-mNH$_2$$^{4+}$** in a buffered solution of 4-cyanoaniline/4-cyanoanilinium in methanol, showing the oxidation (a) and reduction (b) waves. The identified peaks correspond to the cage, with the rest of the peaks arising from electroactivity of the buffer as shown below.

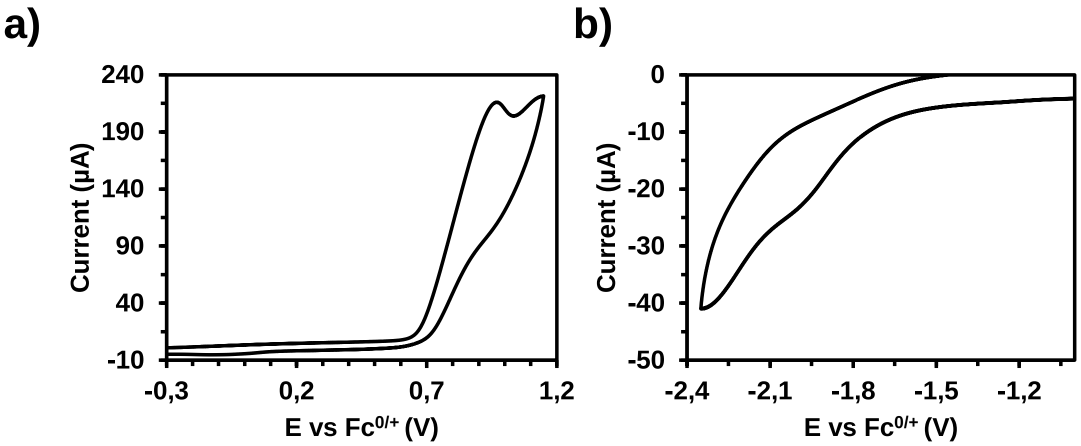

Figure S18. Cyclic voltammetry of a buffered solution of 4-cyanoaniline/4-cyanoanilinium in methanol, showing the oxidation (a) and reduction (b) waves.

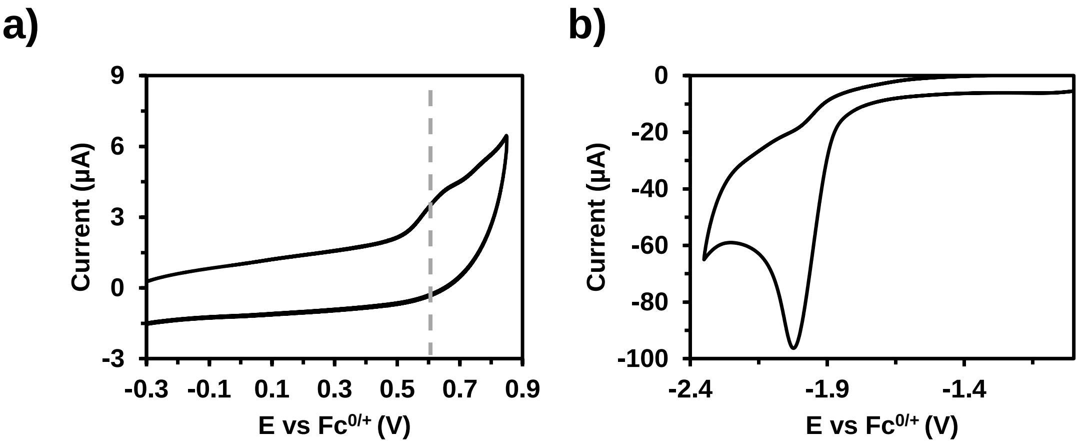

Figure S19. Cyclic voltammetry of **10-mNH$_2$$^{4+}$** in a buffered solution of 2,4,6-collidine/collidinium chloride in methanol, showing the oxidation (a) and reduction (b) waves. The identified peaks correspond to the cage, with the rest of the peaks arising from electroactivity of the buffer as shown below.

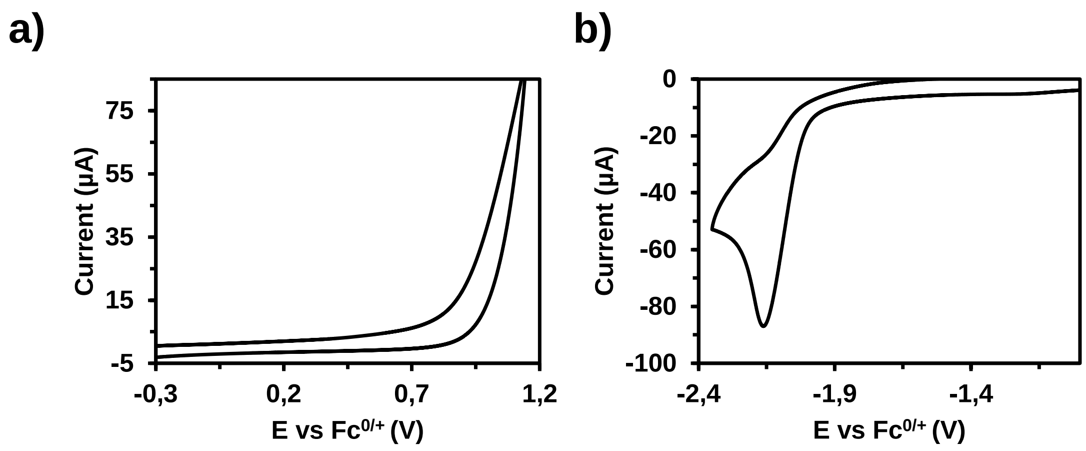

Figure S20. Cyclic voltammetry of a buffered solution of collidine/collidinium chloride in methanol, showing the oxidation (a) and reduction (b) waves.

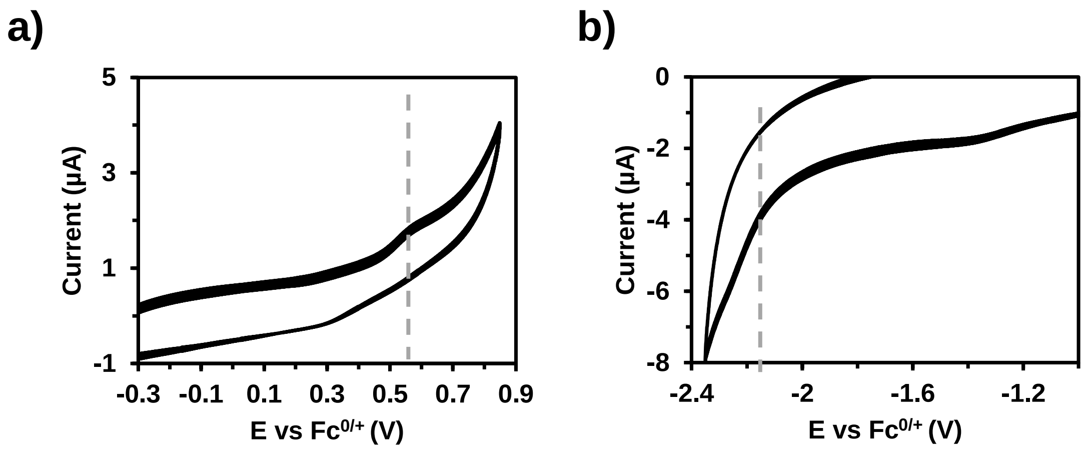

Figure S21. Cyclic voltammetry of **10-mNH$_2^{4+}$** in a buffered solution of Na_2HPO_4/NaH_2PO_4 acid in methanol, showing the oxidation (a) and reduction (b) waves. The decreased intensity of the peaks arises from a lower solubility in this buffer.

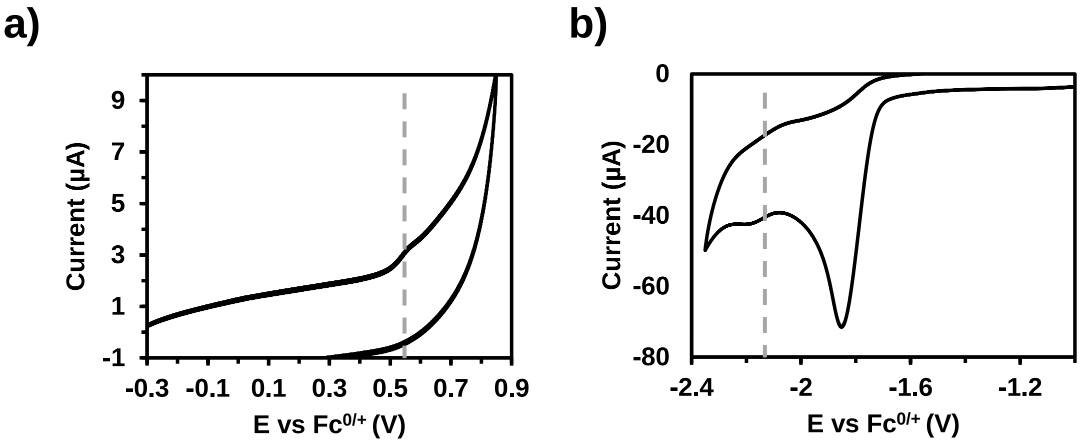

Figure S22. Cyclic voltammetry of **10-mNH$_2^{4+}$** in a buffered solution of pyridine/pyridinium chloride in methanol, showing the oxidation (a) and reduction (b) waves.

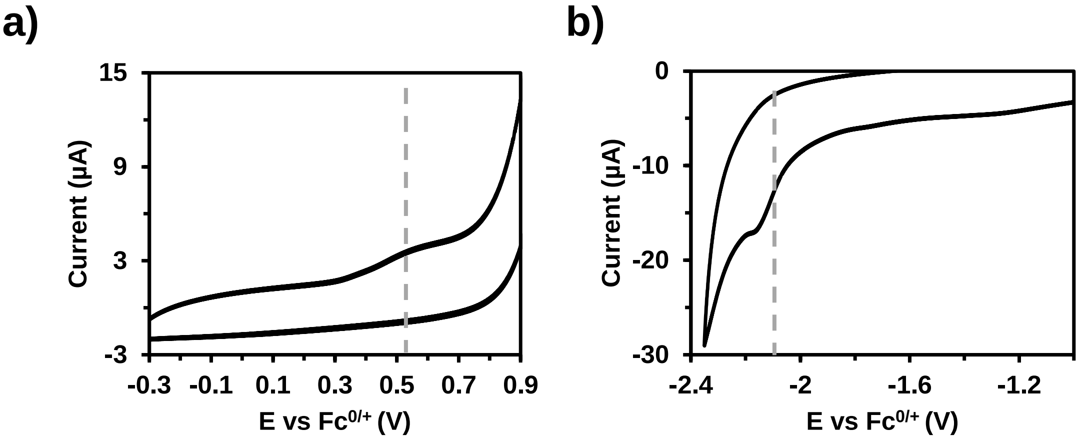

Figure S23. Cyclic voltammetry of **10-mNH$_2^{4+}$** in a buffered solution of triethylamine/triethylamine hydrochloride in methanol, showing the oxidation (a) and reduction (b) waves.

Nature of the redox processes:

We have analysed the electrochemistry of 10-NH_2^{4+} in the presence of different buffers: triethylammonium/triethylamine, 4-cyanoaniline/4-cyanoanilinium chloride, collidine/collidinium chloride, pyridine/pyridinium chloride, Na_2HPO_4/NaH_2PO_4. These buffers did not produce any significant change in the redox potential of the reduction wave, suggesting a bare electron transfer processes. However, the more acidic 4-cyanoanilinium, collidinium, and pyridinium resulted in a large irreversible reduction waves associated to hydrogen evolution at the electrode upon analysis of the buffer. Regarding the oxidation event, a cathodic shift relative to unbuffered solutions is observed, suggesting that the buffer employed can promote proton transfer upon oxidation of the cage. In fact, this shift seems larger with the more basic buffers (e.g., triethylamine or pyridine). Similar observations are made when we employ electrolytes with different proton acceptor character: $NaNO_3$, $NaClO_4$ and KCl. In these cases, the reduction peak barely changes but we observe a cathodic shift of the oxidation event with $NaNO_3$ and $NaClO_4$ (Cl^- oxidation prevents the analysis with KCl) consistent with a coupled proton transfer to the anionic weak bases. Importantly, this feature remains essentially unchanged when we switch to MeCN as a poor proton acceptor solvent using $TBAPF_6$ under unbuffered conditions, supporting that MeOH do not act as proton acceptor. We thus believe that the potential from unbuffered MeOH or MeCN solution is consistent with a bare electron transfer process.

S.3.4. Fluorescence emission

In an Ar-filled glove box, solutions 10^{-2} mM in dried methanol of **1-NH$_2$$^{4+}$**, **10-mNH$_2$$^{4+}$** and 2-amino-[1,1'-biphenyl]-4,4'-dicarboxilic acid were prepared in a fluorescence cuvette, which were covered by a septum and taken out from the glove box in order to measure the fluorescence emission.

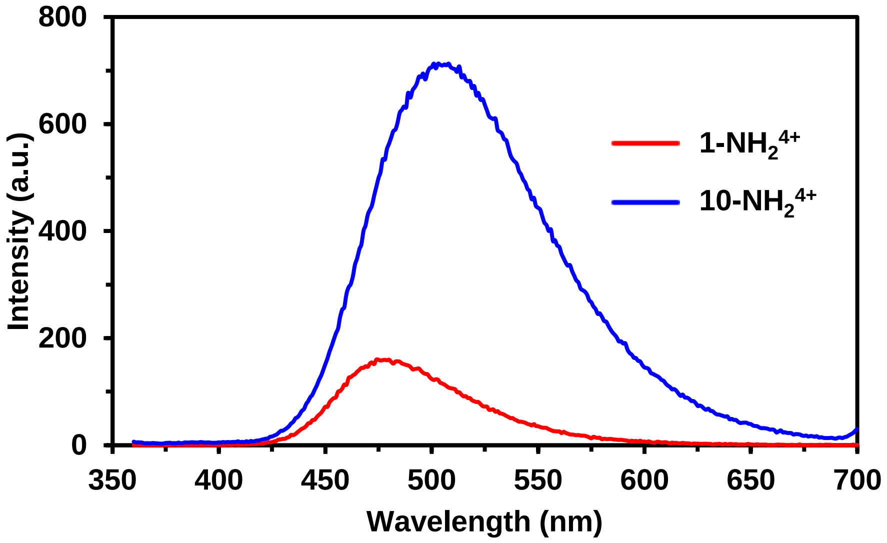

Figure S24. Fluorescence emission spectra of **1-NH$_2$$^{4+}$** and **10-mNH$_2$$^{4+}$** in methanol.

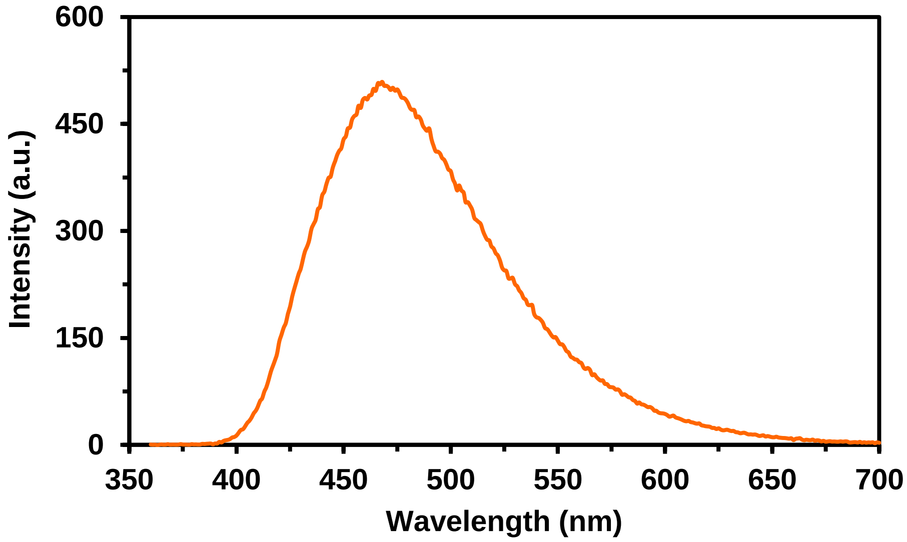

Figure S25. Fluorescence emission spectra of 2-amino-[1,1'-biphenyl]-4,4'-dicarboxilic acid in methanol.

S.3.5. Crystal data

Single crystals of **10-mNH$_2$$^{4+}$** were obtained from the diluted synthesis. In a 25 mL bottle, 20 mg of 2-amino-[1,1'-biphenyl]-4,4'-dicarboxilic acid and 55.5 of dibutylzirconocene dichloride were dissolve in 2 mL of DMF and 1 mL of carbon tetrachloride. Then, 220 µL of H_2O was added drop by drop and the mixture was heated 12 h at 70 ºC. Single crystals were obtained in solution.

10-mNH$_2$$^{4+}$ data were collected at 120 K on a Bruker D8 VENTURE PHOTON III-14 diffractometer, employing graphite monochromatized Mo-Kα (λ = 0.71073 Å) radiation. Multi-scan absorption corrections were applied using the SADABS[10] routine. The structures were solved by direct methods with SHELXT-2014[3] and refined with SHELXL-2018[11] using OLEX 2-1.3.[12] All the non-hydrogen atoms were refined by full-matrix least-squares techniques. Due to positional disorder, some atoms of cyclopentadienyl rings were split, while it was not possible to completely model the *n*-butyl chain. The carbon atoms of the *n*-butyl chains and of some of the cyclopentadienyl rings were not assigned anisotropic displacement parameters. The solvent molecules which occupy the central cavity were found highly disordered and any attempts to describe the smeared electronic density belonging to them were unsuccessful. Contributions to diffraction due to these highly disordered molecules as well as the non-modelled *n*-butyl chains (see above) were removed using the SQUEEZE routine of PLATON;[13] the crystal structures were then refined again using the generated *hkl* data.

Final R_1 = 0.0917, wR_2 = 0.2844, *GOOF* = 1.079, for **10-mNH$_2$$^{4+}$**.

Crystal data and refinement results are shown in Table S2, while selected bond distances and angles are collected in Table S3. The CIF files has been deposited in the Cambridge Crystallographic Data Centre (CCDC) under deposition number 2420538.

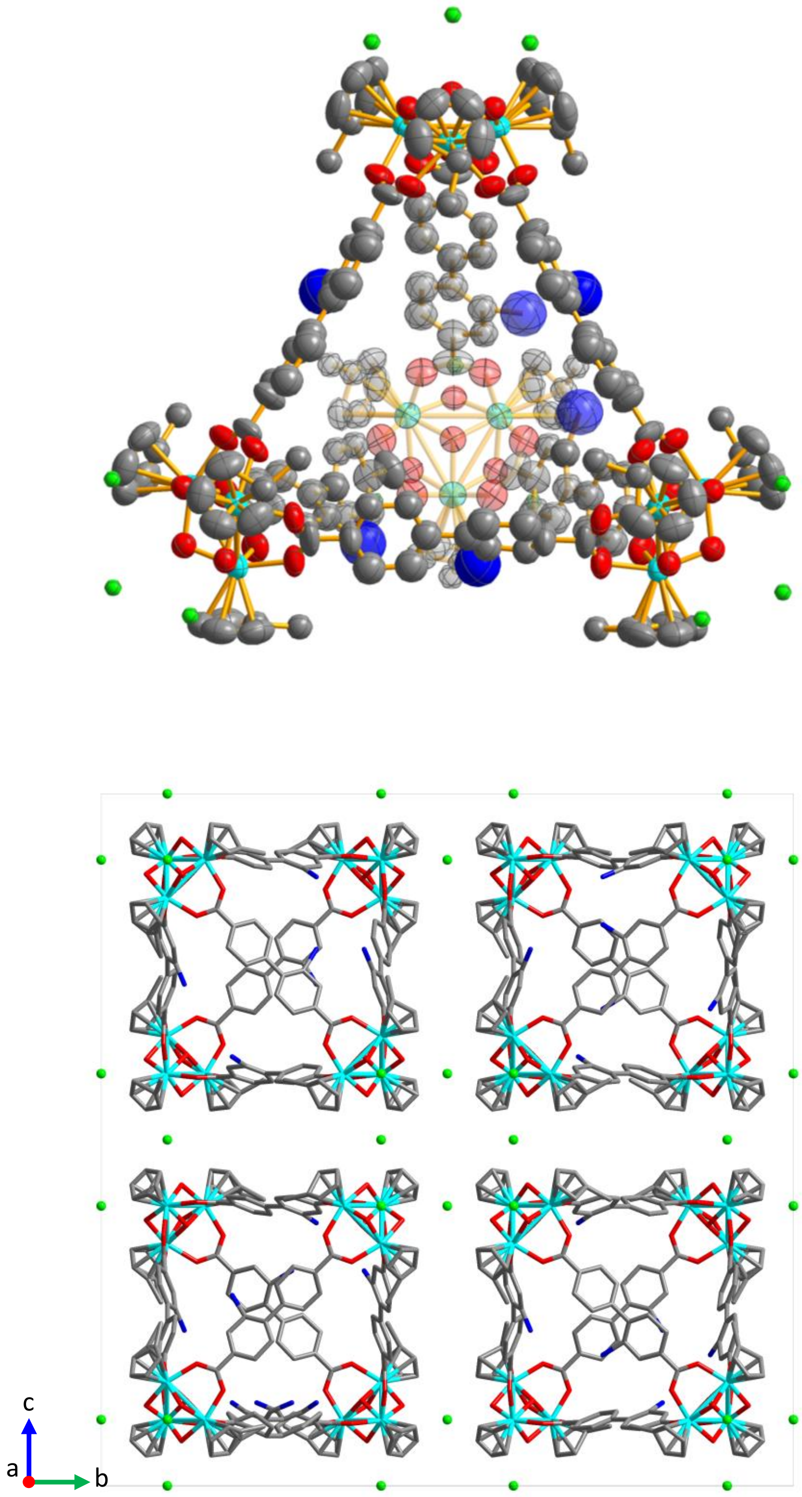

Figure S26. Top: Representation of one tetrahedron. Ellipsoids depicted with 50% probability level. Bottom: Crystal structure of **10-mNH$_2$$^{4+}$** viewed along the [100] crystallographic direction. Horizontal axis, *b*; vertical axis, *c*. Ligand and NH_2 groups have been ordered for clarity. Atom colour code: carbon, grey; chlorine, green; nitrogen, blue; oxygen, red; zirconium, light blue.

Table S2. Crystal data and refinement results for **10-mNH$_2$$^{4+}$**.

**Empirical formula**	$C_{150}Cl_4N_6O_{40}Zr_{12}$
**Formula weight**	3833
**Temperature/K**	120(2)
**Crystal system**	Cubic
**Space group**	*Fm-3m*
**a/Å**	42.6196(9)
**b/Å**	42.6196(9)
**c/Å**	42.6196(9)
**α/°**	90
**β/°**	90
**γ/°**	90
**Volume/Å^3**	77416(5)
**Z**	8
**ρ_{calc}g/cm^3**	0.646
**μ/mm^{-1}**	0.381
**F(000)**	14480.0
**Crystal size/mm^3**	0.216 × 0.196 × 0.176
**Radiation**	MoKα (λ = 0.71073)
**2Θ range for data collection/°**	3.822 to 46.53
**Index ranges**	-44 ≤ h ≤ 47, -47 ≤ k ≤ 35, -47 ≤ l ≤ 19
**Reflections collected**	49894
**Independent reflections**	2760 [R_{int} = 0.0606, R_{sigma} = 0.0375]
**Data/restraints/parameters**	2760/3/98
**Goodness-of-fit on F^2**	1.072
**Final R indexes [I>=2σ (I)]**	R_1 = 0.0917, wR_2 = 0.2842
**Final R indexes [all data]**	R_1 = 0.1214, wR_2 = 0.3393
**Largest diff. peak/hole / e Å^{-3}**	0.57/-0.75

Table S3. Selected bond distances and angles of **10-mNH$_2^{4+}$**.

**Atoms**	**Length (Å)/Angle (°)**	**Atoms**	**Length (Å)/Angle (°)**
$Zr^{(1)}$ - $O^{(1)2}$	2.138(4)	$O^{(1)}$ - $Zr^{(1)}$ - $C^{(8)}$	151.7(3)
$Zr^{(1)}$ - $O^{(2)}$	2.075(4)	$O^{(1)}$ - $Zr^{(1)}$ - $C^{(10)}$	153.8(4)
$Zr^{(1)}$ - $O^{(3)3}$	2.205(19)	$O^{(1)}$ - $Zr^{(1)}$ - $C^{(9)3}$	152.5(3)
$Zr^{(1)}$ - $Zr^{(1)1}$	3.3524(19)	$O^{(1)}$ - $Zr^{(1)}$ - $C^{(8)3}$	153.8(4)
$Zr^{(1)}$ - $C^{(9)}$	2.523(10)	$O^{(1)}$ - $Zr^{(1)}$ - $C^{(7)}$	79.0(3)
$Zr^{(1)}$ - $C^{(10)}$	2.526(14)	$C^{(8)}$ - $Zr^{(1)}$ - $C^{(10)}$	54.0(4)
$Zr^{(1)}$ - $C^{(8)}$	2.502(10)	$C^{(8)3}$ - $Zr^{(1)}$ - $C^{(9)}$	51.1(4)
$O^{(3)}$ - $C^{(1)}$	1.274(8)	$C^{(8)3}$ - $Zr^{(1)}$ - $C^{(9)3}$	30.8(4)
$O^{(1)}$ - $Zr^{(1)}$ - $O^{(1)2}$	73.7(2)	$C^{(10)}$ - $Zr^{(1)}$ - $C^{(8)3}$	48.7(4)
$O^{(1)}$ - $Zr^{(1)}$ - $O^{(1)2}$	92.1(4)		
Symmetry transformations used to generate equivalent atoms: ^{1} 1/2 -z, +x, 1/2-y; ^{2} +y, 1/2-z, 1/2-x; ^{3} 1/2-z, +y, 1/2-x; ^{4} +x, 1/2-z, 1/2-y; ^{5}+x, 1/2-y, 1/2-z			

S.4. Lifetime measurements

In an Ar-filled glove box, solutions in dried methanol or acetonitrile 10^{-2} mM of **10-mNH$_2^{4+}$** were prepared in a fluorescence cuvette, which were covered by a septum and taken out from the glove box.

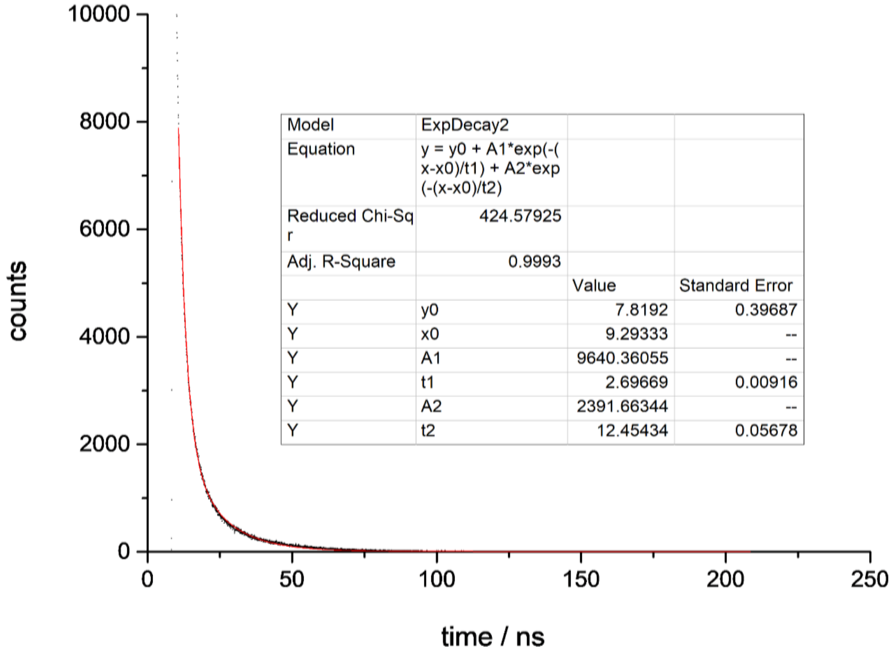

Figure S27. Lifetime decays of the excited state of **10-mNH$_2^{4+}$** in MeOH.

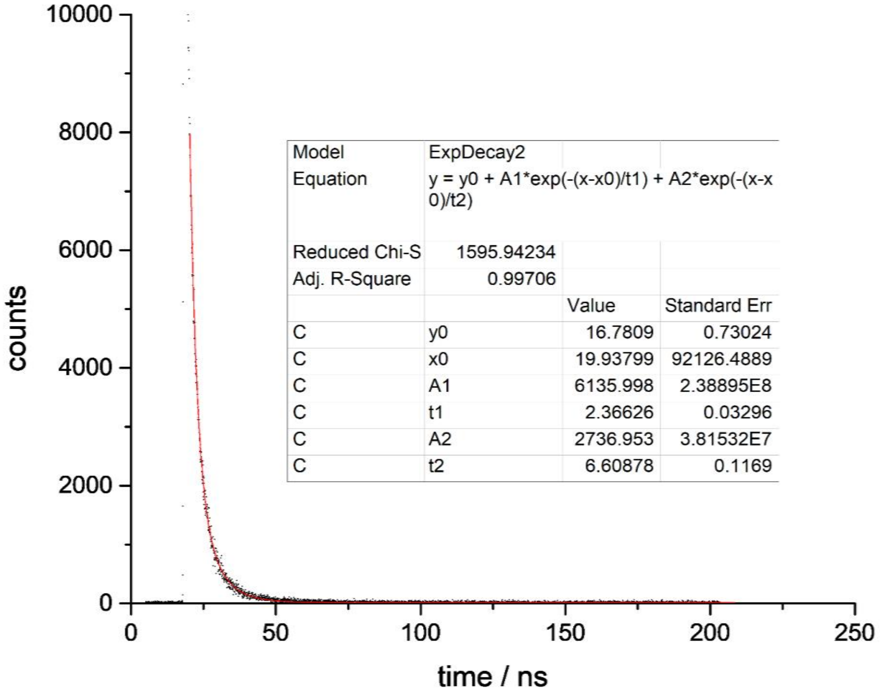

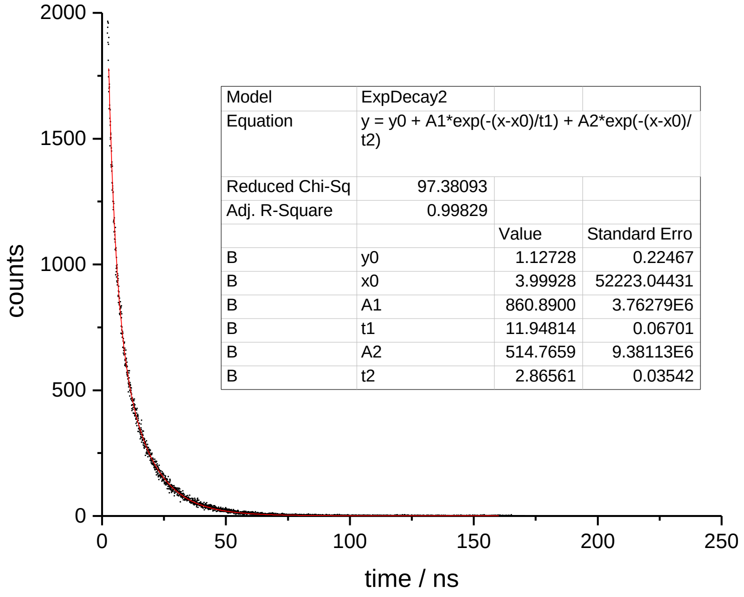

Figure S28. Lifetime decays of the excited state of **10-mNH$_2$$^{4+}$** (top) and **1-NH$_2$$^{4+}$** (down) in MeCN.

S.5. Stern-Volmer

In an Ar-filled glove box, 2.2 mg of **10-mNH$_2^{4+}$** (0,5 µmol) were dissolved in 4 mL of dry methanol. Then, 2 mL of the solution were put in a fluorescence cuvette and the other 2 mL were added to 1000 equivalents of acetophenone. The cuvette and the solution were covered by a septum and taken out from the glove box in order to measure the fluorescence emission.

Stern-Volmer plots were made by monitoring the relationships between the initial emission intensity without quencher (I_0) and the emission intensity (I) versus the quencher concentration (Q). K_{SV} is the slope of the plot corresponding to the product between the kinetic rate of the quenching process (PCET in our case, k_{PCET}) and the lifetime of the excited state (τ_0). The resulting plot was fitted to a linear regression, from which K_{SV} can be extracted.

$$\frac{I_0}{I} = 1 + K_{SV}[Q] \quad Eq.S1$$

$$K_{SV} = k_{PCET}\tau_0 \quad Eq.S2$$

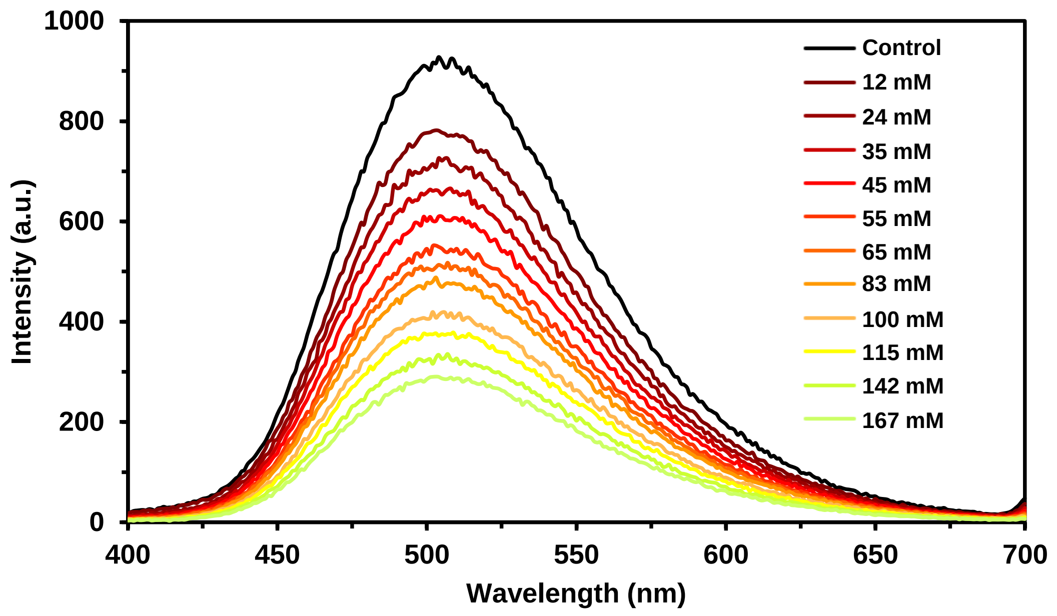

Figure S29. Fluorescence emission spectra of **10-mNH$_2^{4+}$** (0.2 mM) in methanol in the presence of increasing concentration of acetophenone. The excitation wavelength was 350 nm.

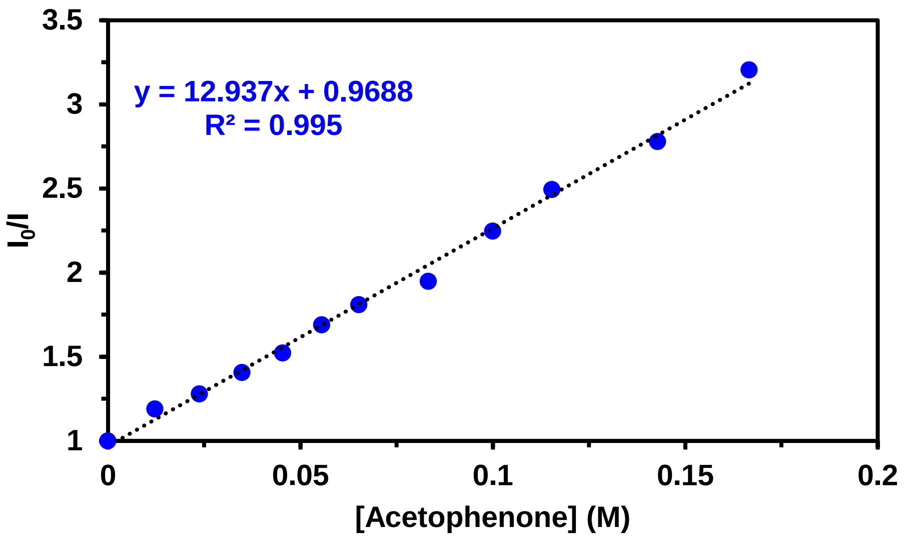

Figure S30. Stern-Volmer plot of I_0/I versus quencher concentration (acetophenone).

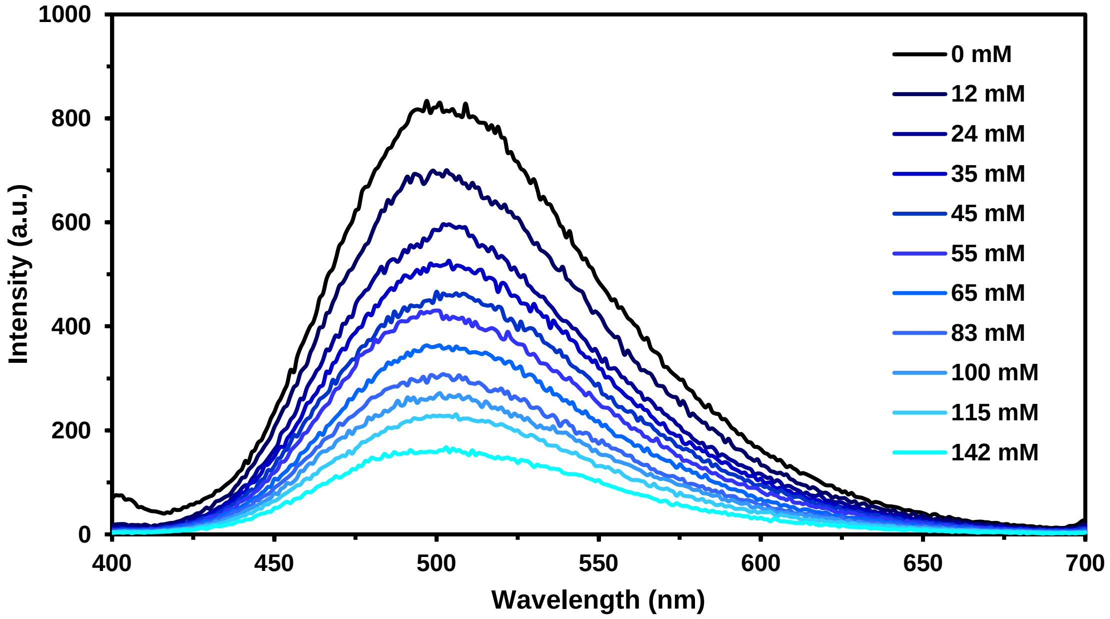

Figure S31. Fluorescence emission spectra of **10-mNH$_2^{4+}$** (0.2 mM) in acetonitrile in the presence of increasing concentration of acetophenone. The excitation wavelength was 350 nm.

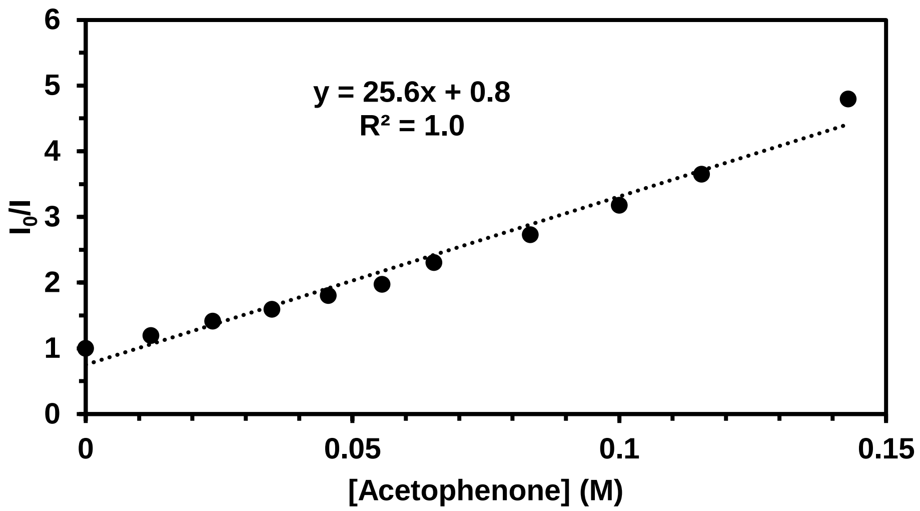

Figure S32. Stern-Volmer plot of I_0/I versus quencher concentration (acetophenone).

S.6. Quantum yield of the photochemical PCET reaction

The relative quantum yield was determined through the potassium ferrioxalate chemical actinometry method.[14] $K_3Fe(C_2O_4)_3$ was prepared and used in solution by making a 10 mM Fe_2SO_4 and 60 mM $K_2C_2O_4$ stock solution of 4 mL in 4% H_2SO_4 (aq). 3 mL of this solution was irradiated at 440 nm for 5 seconds. Care was taken to minimize light exposure between irradiation cycles.

A 0.2% by weight solution of 1,10-phenanthroline in 1 mL of water and a 0.6 M NaOAc buffer in 1 mL of water with 1% H_2SO_4 (aq) were prepared separately. A 100 µL aliquot of the irradiated solution was placed into a 10 mL volumetric flask along with 200 µL of the phenanthroline solution and 50 µL of buffer, and the solution was diluted with water. The complexation of Fe^{2+} with 1,10-phenanthroline resulted in a bright red solution that had a characteristic absorption at 510 nm. 2 mL of the solution were put in a cuvette and were measure by UV-visible absorption spectra. For the control, these steps were repeated with a 100 µL aliquot of non-irradiated solution.

$$2Fe^{3+} + C_2O_4^{2-} \xrightarrow{hv} 2Fe^{2+} + 2CO_2$$

Because full conversion was not reached after 5 s, but rather sometime between 5 and 10 s, the run at 5 s was chosen for analysis. These gave an absorbance of 0.33 at 510 nm vs. control.

$$I\ (\mathrm{mol} \cdot \mathrm{min}^{-1}) = \frac{AV_2V_3}{\varepsilon b \phi_\lambda t V_1} \quad Eq.S3$$

Where,

A = absorbance at 510 nm

V_2 = volume of actinometer irradiated (2 mL)

V_3 = final volume of quantified sample (10 mL)

ε = extinction coefficient of ferrous 1,10-phenanthroline at 510 nm ($\sim 1.11 \cdot 10^4\ M^{-1} \cdot cm^{-1}$)

b = path length of cuvette (1 cm)

ϕ_λ = quantum yield of ferrous production at 436 nm (~1.11)

t = time of irradiation (5 s)

V_1 = volume of aliquot of irradiated sample taken (100 µL)

Giving a light intensity in mol of photons per unit time. Using the gathered absorbance data, a light intensity of 6,12 · 10^{-5} mol · min^{-1} was obtained, and this photon flux was used to calculate relative quantum yield for a given substrate yield over a known irradiation time.

A photocatalytic reaction was carried out in the same cuvette as used for measuring the light intensity. **10-mNH$_2^{4+}$** (1 µmol) and acetophenone (50 µmol) were solved in 2 mL of isopropanol. The mixture was stirred and irradiated with visible light (440 nm wavelength, 100 W KESSIL LIGHT LEDs) for 10 minutes. Then, the product was analysed by GC according to previous calibration curve.[8]

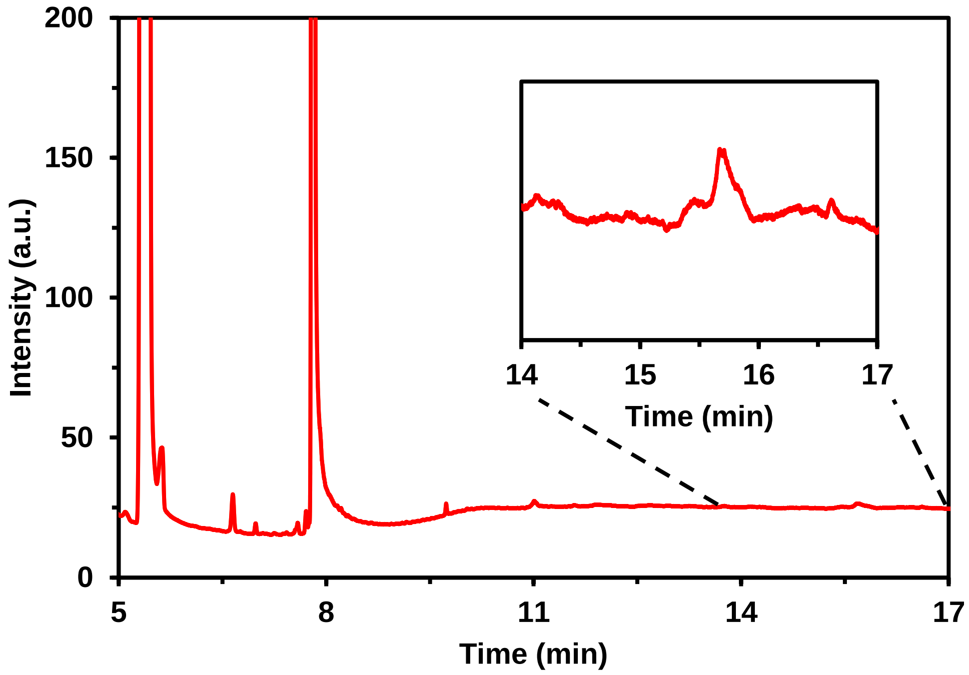

Figure S33. GC measurement of the reaction in iPrOH (catalytic conditions).

S.7. Photocatalytic reactions

General protocol:

The photocatalytic reactions were carried out under nitrogen atmosphere. In a 5 mL glass vial **1-NH$_2^{4+}$** or **10-mNH$_2^{4+}$** (1 µmol), substrate (50 µmol), H_2O (3000 µmol), TEA (1000 µmol) and solvent (2 mL) were added depending on the specific conditions. The mixture was degassed during 15 mins and irradiated with blue light (440 nm wavelength, 100 W KESSIL LIGHT LEDs) under stirring during 24h. Then, the solvent was evaporated and the solid powder was dissolved in 800 µL of deuterated methanol or chloroform with an internal standard, dried over sodium sulphate and analysed by ^{1}H-NMR.

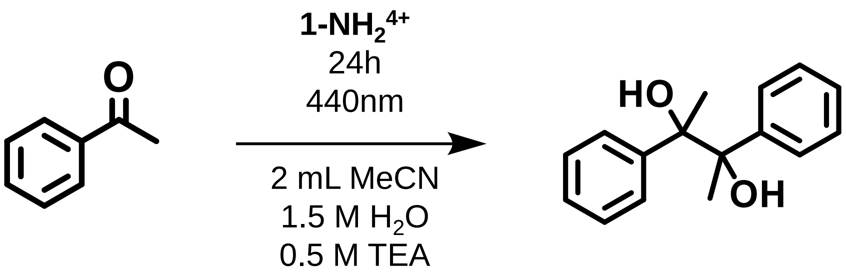

Figure S34. Scheme of the photocatalytic reaction mediated by **$1-NH_2^{4+}$** in MeCN with H_2O and TEA.

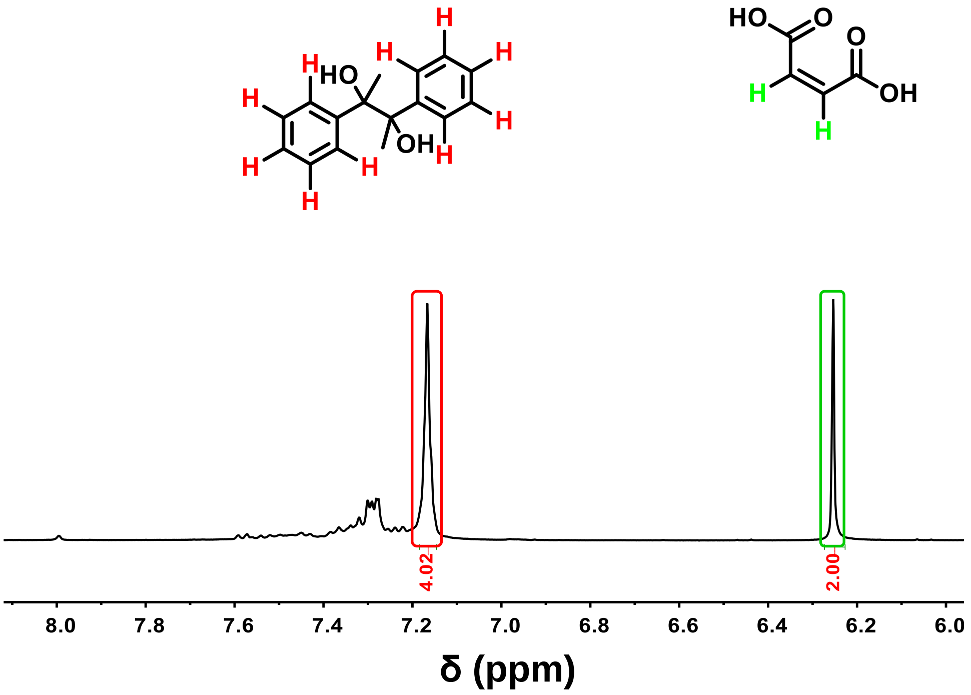

Figure S35. ^1H-NMR spectra in MeOD of the photocatalytic reaction with **$1-NH_2^{4+}$** showing the product (red) and the internal standard signal (maleic acid, green).

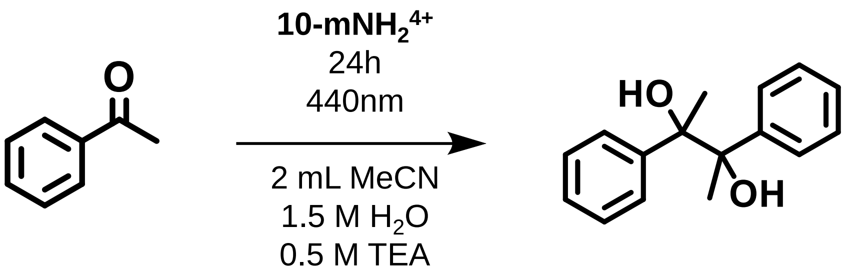

Figure S36. Scheme of the photocatalytic reaction mediated by **10-mNH$_2$$^{4+}$** in MeCN with H_2O and TEA.

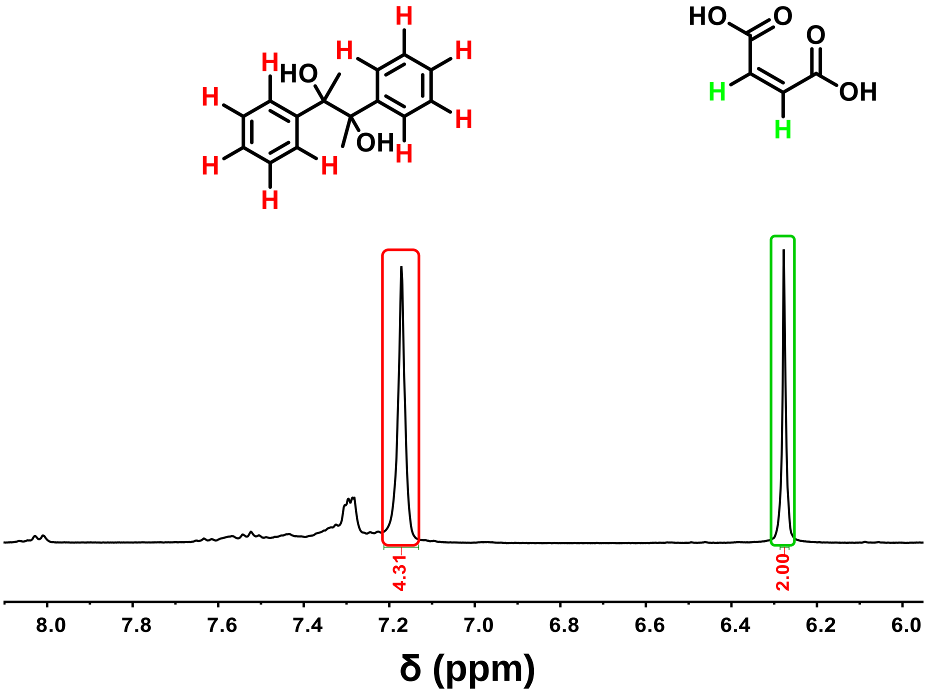

Figure S37. ^{1}H-NMR spectra in MeOD of the photocatalytic reaction with **10-mNH$_2$$^{4+}$** showing the product (red) and the internal standard signal (maleic acid, green).

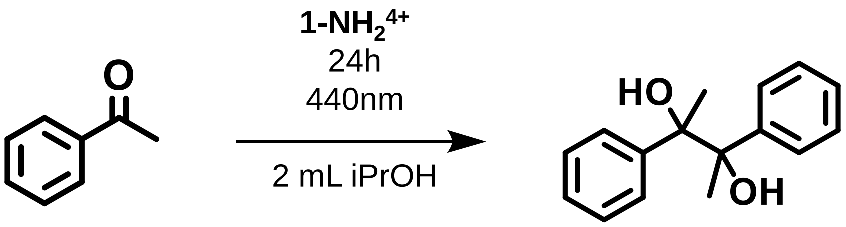

Figure S38. Scheme of the photocatalytic reaction mediated by **1-NH$_2^{4+}$** in iPrOH with H_2O and **25 equivalents** of acetophenone.

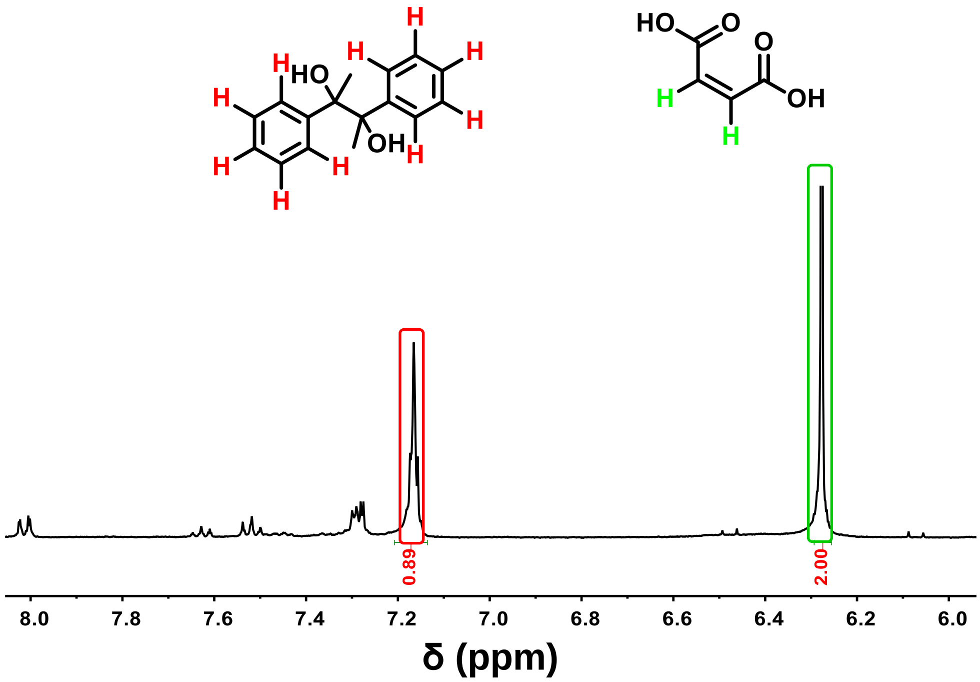

Figure S39. ^{1}H-NMR spectra in MeOD of the photocatalytic reaction with **1-NH$_2^{4+}$** showing the product (red) and the internal standard signal (maleic acid, green).

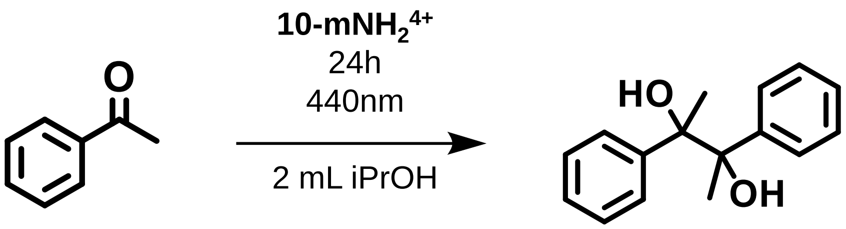

Figure S40. Scheme of the photocatalytic reaction mediated by **10-mNH$_2$$^{4+}$** in iPrOH with H_2O and **25 equivalents** of acetophenone.

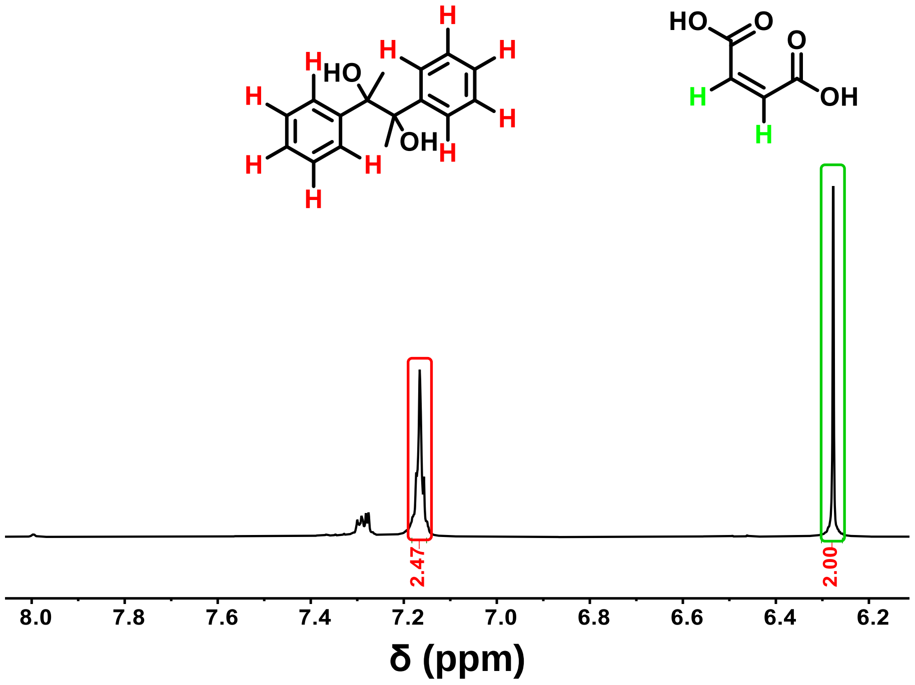

Figure S41. ^{1}H-NMR spectra in MeOD of the photocatalytic reaction with **10-mNH$_2$$^{4+}$** showing the product (red) and the internal standard signal (maleic acid, green).

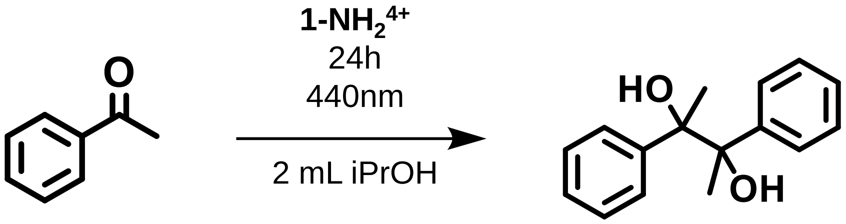

Figure S42. Scheme of the photocatalytic reaction mediated by **1-NH$_2$$^{4+}$** in iPrOH with H_2O and **50 equivalents** of acetophenone.

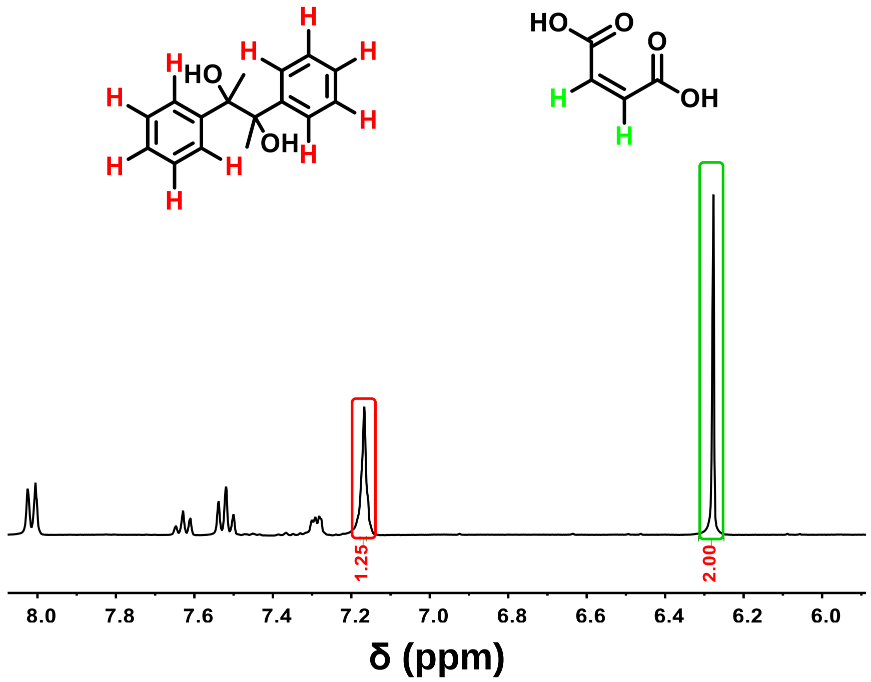

Figure S43. ^{1}H-NMR spectra in MeOD of the photocatalytic reaction with **1-NH$_2$$^{4+}$** showing the product (red) and the internal standard signal (maleic acid, green).

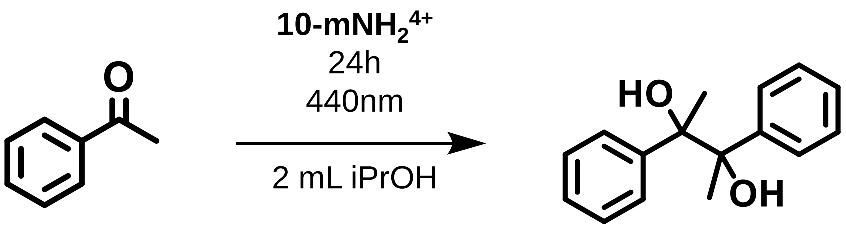

Figure S44. Scheme of the photocatalytic reaction mediated by **10-mNH$_2$$^{4+}$** in iPrOH with H_2O and **50 equivalents** of acetophenone

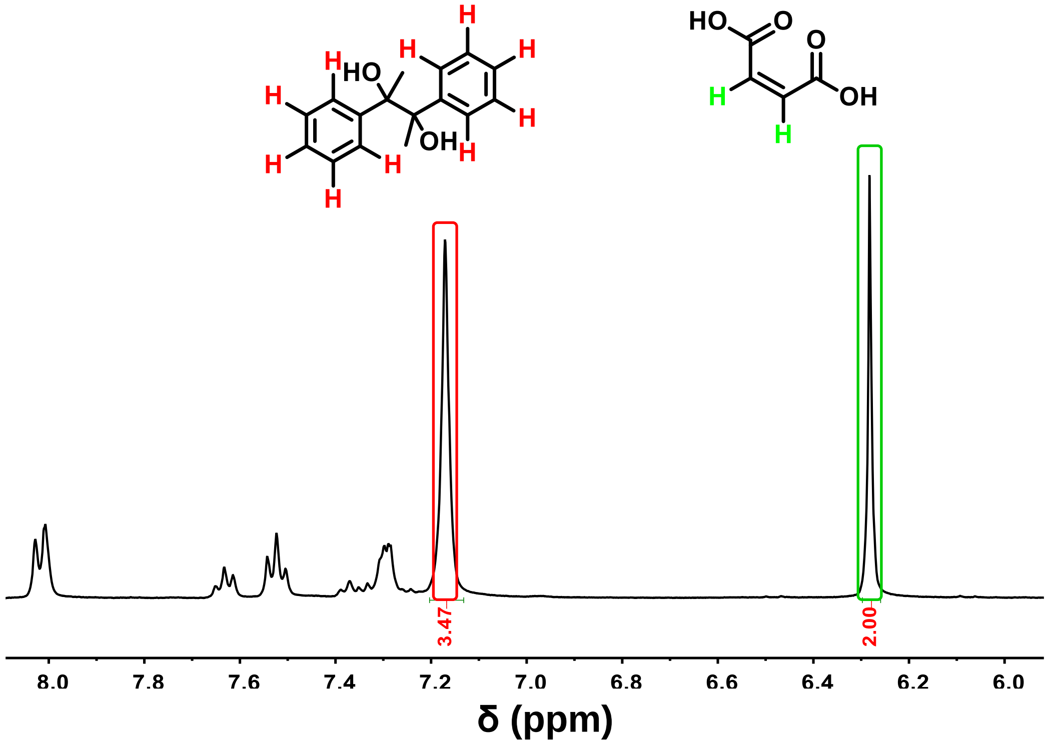

Figure S45. ^{1}H-NMR spectra in MeOD of the photocatalytic reaction with **10-mNH$_2$$^{4+}$** showing the product (red) and the internal standard signal (maleic acid, green).

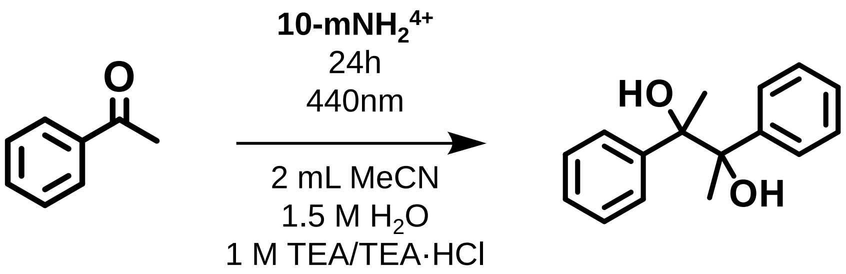

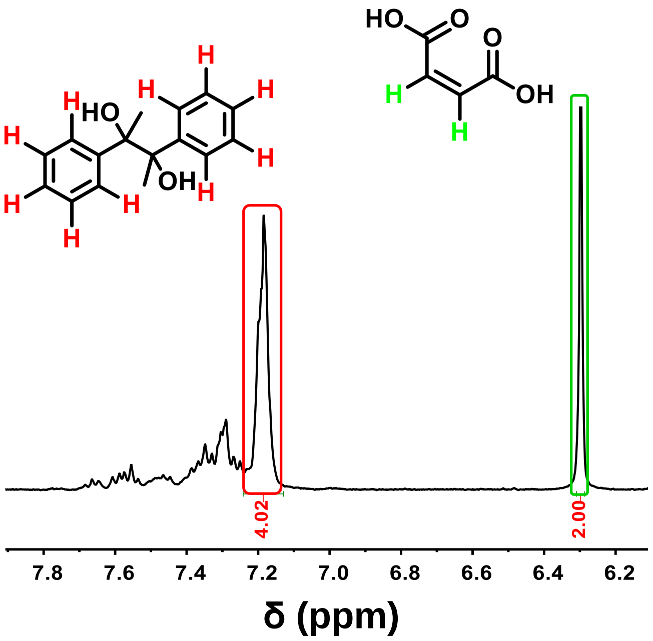

Figure S46. ^{1}H-NMR spectra in MeOD of the photocatalytic reaction with **10-mNH$_2$$^{4+}$** showing the product (red) and the internal standard signal (maleic acid, green).

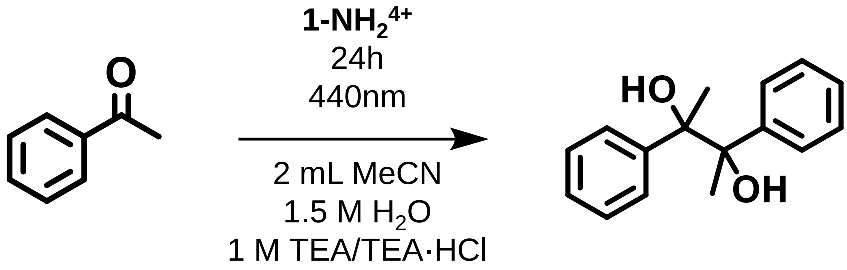

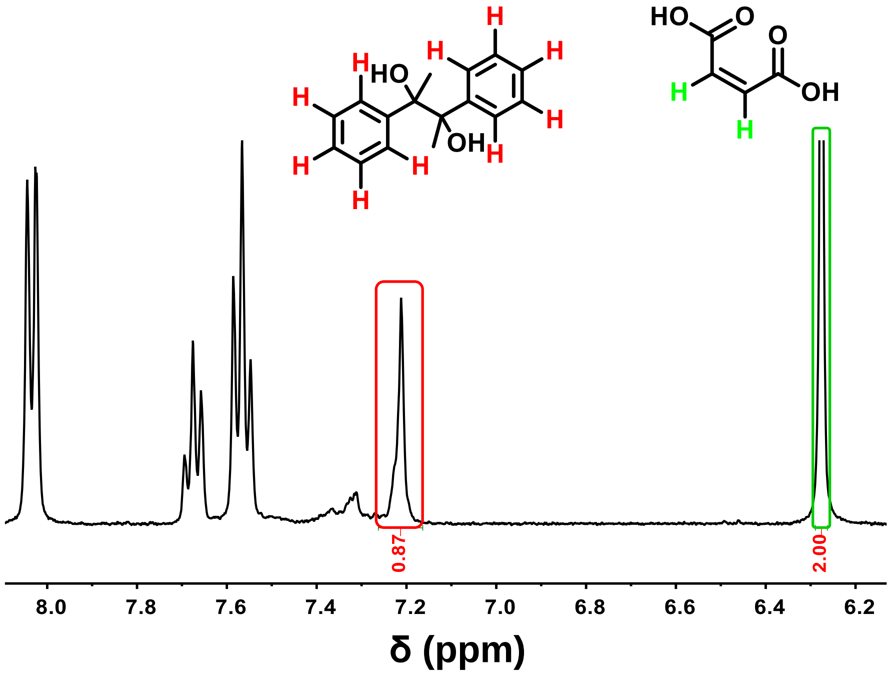

Figure S47. ^{1}H-NMR spectra in MeOD of the photocatalytic reaction with **10-mNH$_2^{4+}$** showing the product (red) and the internal standard signal (maleic acid, green).

S.8. DFT calculations

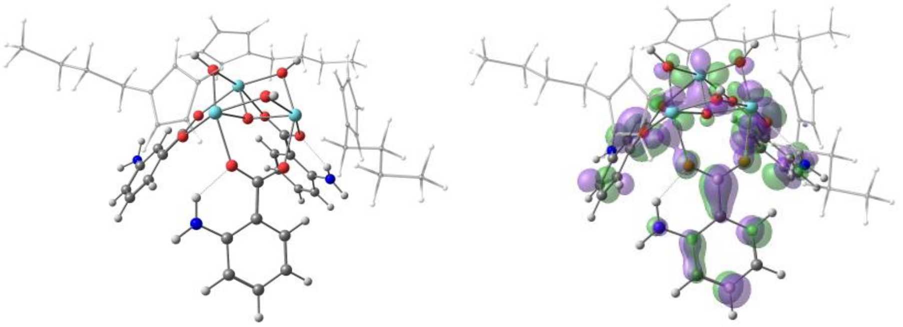

Figure S48. DFT optimized structure of **1-NH$_2^{4+}$** in the triplet excited state and SOMO.

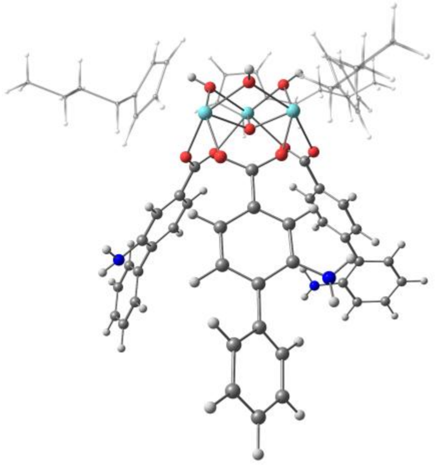

Figure S49. DFT optimized structure of **10-mNH$_2^{4+}$** in the singlet ground state.

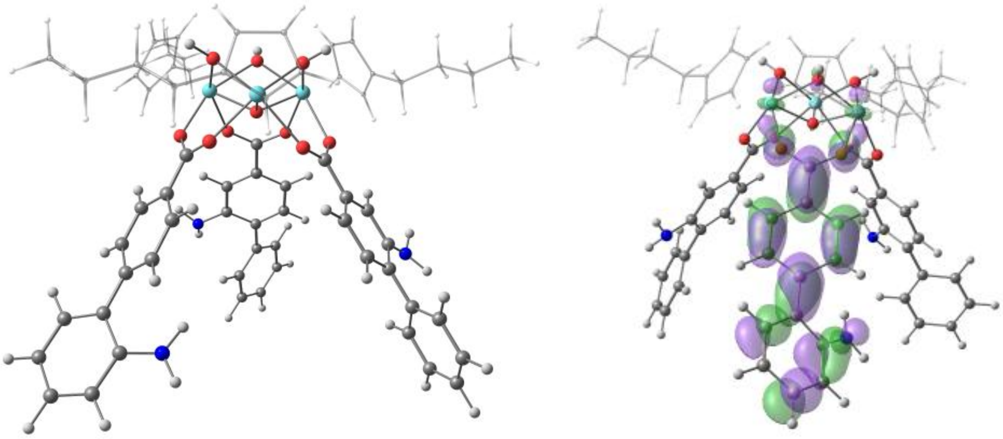

Figure S50. DFT optimized structure of **10-mNH$_2^{4+}$** in the triplet excited state and SOMO.

TD-DFT

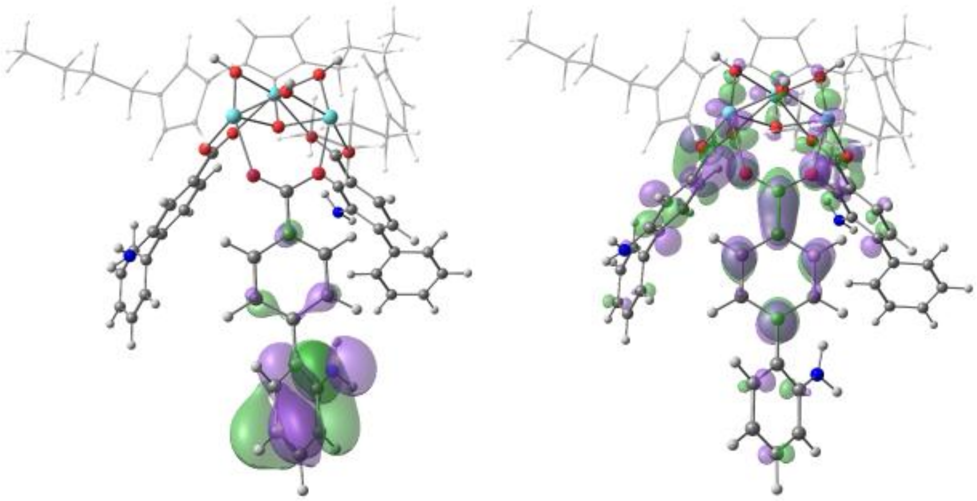

Figure S51. HOMO (left) and LUMO (right) involved in the transition at 520 nm.

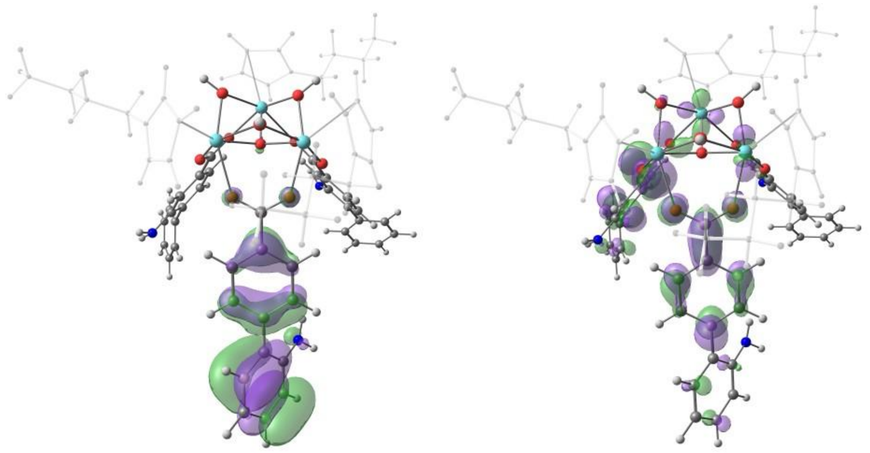

Figure S52. Donor (left) and acceptor (right) orbitals in the transition at 321 nm.

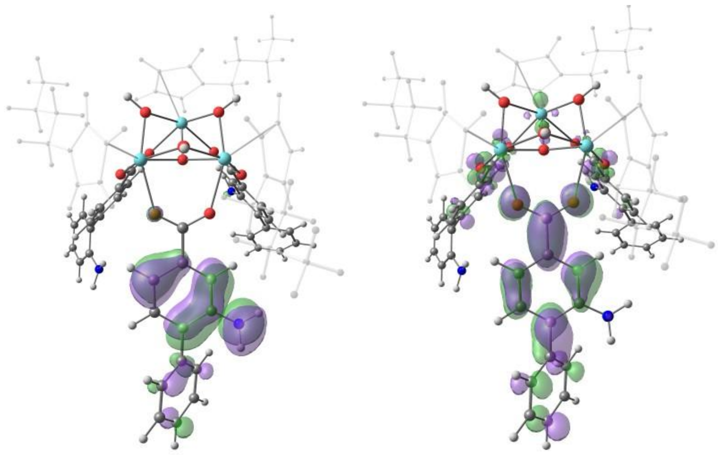

Figure S53. Donor (left) and acceptor (right) orbitals in the transition at 350 nm.

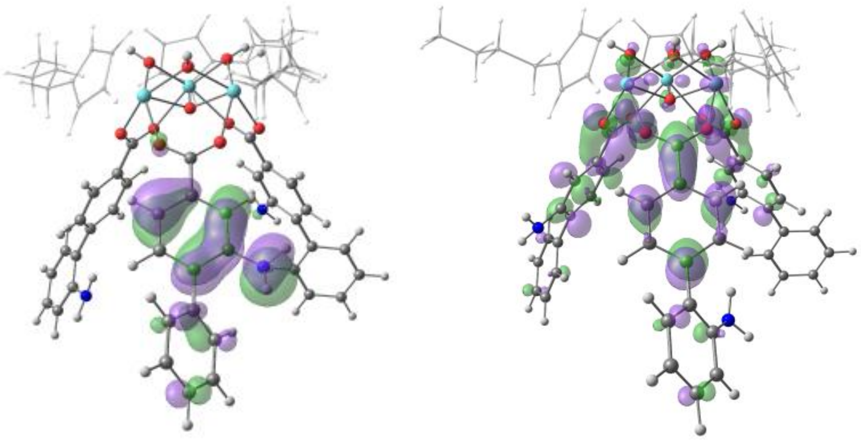

Figure S54. Donor (left) and acceptor (right) orbitals in the transition at 410 nm.

<u>**XYZ coordinates**</u>

10-mNH$_2^{4+}$ (singlet)

E = -14095.40082130 H

Zr	0	6.34803400	14.60080100	14.37447900
O	0	4.89293200	13.62353200	15.64247300
O	0	5.14104100	13.37788700	13.15803200
O	0	7.46621900	14.89875600	12.42285000
C	0	7.06043300	14.84909100	11.21158700
C	0	8.10387800	14.77506600	10.13656100
C	0	7.71922300	14.70500600	8.78795300
C	0	6.62277100	15.84507200	16.72866400
C	0	8.59174700	15.51814300	15.59417900
H	0	9.54386000	15.09263100	15.26417300
C	0	7.77926700	15.00607800	16.65468300
C	0	10.06329500	14.60185000	8.08383800
C	0	8.67055900	14.61822100	7.74484400
O	0	5.83434800	14.87069300	10.85452700
C	0	9.47483600	14.77600800	10.46972200
C	0	10.42339100	14.69611000	9.44635600
C	0	6.72451900	16.85924100	15.73197100
C	0	7.94922600	16.65897900	15.03266200
C	0	2.98720600	11.65520800	16.74398000
H	0	3.18172400	12.44969400	17.46842800
C	0	3.13435300	9.70038000	15.54019700
H	0	3.50255900	8.75244700	15.13761200
C	0	3.77399300	10.48538100	16.54242700
C	0	2.28232600	16.62564000	11.75648700
H	0	2.27414900	17.31848300	12.60210000
C	0	2.77963700	15.63665300	9.74296600
H	0	3.25208100	15.41747700	8.78122300
C	0	3.09865200	16.74267900	10.58867300
C	0	1.86016600	11.58293100	15.86979500
H	0	1.05684500	12.32144700	15.81145900
C	0	1.94267100	10.36595700	15.12210000

C	0	1.46399600	15.46437500	11.62218900
H	0	0.71023200	15.12685400	12.33687900
C	0	1.77262800	14.85294500	10.37473800
Zr	0	3.87620500	14.55383900	11.98734800
O	0	4.76696500	15.87322400	13.51406000
O	0	3.73882200	12.53981600	10.83716000
C	0	3.75271700	11.32389900	11.21644700
C	0	3.86060500	10.26018300	10.16683400
C	0	3.93348600	8.90033000	10.53438700
C	0	4.06120400	8.22837300	8.18102600
C	0	4.03178400	7.90396100	9.55995400
O	0	3.66029900	10.93426900	12.43251400
C	0	3.88525600	10.59434000	8.79725600
C	0	3.98158700	9.59593900	7.82329300
Zr	0	3.99568400	12.07196500	14.37008800
O	0	2.65678200	13.59588500	13.56121900
O	0	5.99860000	10.94860000	14.50099200
C	0	7.20277700	11.36727700	14.43918300
C	0	8.28184000	10.32576600	14.36751800
C	0	9.62949700	10.70641300	14.27229700
C	0	10.33090200	8.35991600	14.28552000
C	0	10.67182300	9.75049800	14.22362300
O	0	7.54507800	12.59308500	14.45942100
C	0	7.94623000	8.95683900	14.42138800
C	0	8.96790800	8.00516400	14.38796100
C	0	3.40411100	19.07429000	9.61275800
H	0	2.88877600	18.74260000	8.68986300
H	0	2.61533800	19.46714100	10.28424700
C	0	4.39336800	20.19903800	9.27125900
H	0	5.17530100	19.80266300	8.59351100
H	0	4.91888800	20.51441000	10.19456900
C	0	3.72556100	21.41742200	8.62352100
H	0	4.46469400	22.20599500	8.39197600
H	0	3.22114700	21.14415100	7.67768600

H	0	2.96275000	21.85988100	9.29149500
C	0	8.10073300	13.84786700	17.56653800
H	0	7.17016900	13.29709900	17.80374100
H	0	8.76356900	13.13737800	17.03995900
C	0	8.77418900	14.28096600	18.88785000
H	0	9.71193700	14.82307100	18.65577500
H	0	8.11758800	15.00493900	19.40941000
C	0	9.08084400	13.10081300	19.82265300
H	0	8.13900500	12.56149400	20.04620300
H	0	9.72945800	12.37645300	19.29129400
C	0	9.75241300	13.51979800	21.13557300
H	0	9.95983900	12.64411100	21.77749200
H	0	10.71429500	14.03388100	20.95015900
H	0	9.11109600	14.21208400	21.71296300
C	0	0.93750300	9.82058300	14.13262300
H	0	1.46590600	9.17970600	13.40241900
H	0	0.24772300	9.14251200	14.67723300
C	0	0.09725900	10.86815500	13.38314200
H	0	-0.46569700	11.48385500	14.11145000
H	0	0.77023700	11.56103800	12.84144900
C	0	-0.89061200	10.24031900	12.38690200
H	0	-0.32616900	9.64026700	11.64598200
H	0	-1.54440100	9.52594300	12.92543700
C	0	-1.75697300	11.27103600	11.65489400
H	0	-2.36380200	11.86405800	12.36487400
H	0	-1.13615500	11.97991600	11.07475700
H	0	-2.45242900	10.78246700	10.94826300
H	0	1.32857800	13.93522500	9.98153400
H	0	5.79784900	15.73063100	17.43748800
H	0	8.32030000	17.25945500	14.19802300
H	0	5.99896700	17.65604200	15.54986700
H	0	4.71032400	10.24327400	17.05112400
H	0	3.90501200	8.62771200	11.59355800
H	0	4.01044200	9.87669400	6.76486900

H	0	4.05968700	6.85441300	9.87319100
H	0	9.88576600	11.77068100	14.24389700
H	0	6.65490700	14.72054800	8.53169400
H	0	11.48824900	14.67977900	9.70410200
C	0	4.07389700	17.85244000	10.28048900
H	0	4.86928000	17.46838700	9.61562800
H	0	4.57060900	18.18071100	11.21325200
H	0	1.68751700	13.50423700	13.53584800
H	0	5.00489500	13.52806300	16.60580200
H	0	6.89874500	8.65108100	14.48948500
H	0	4.86778900	16.83658100	13.40212400
C	0	4.11736000	7.16892500	7.13510300
C	0	3.18778500	7.20179200	6.07194100
C	0	5.08121200	6.10820800	7.17485700
C	0	3.14911900	6.21177200	5.08236100
H	0	2.45383500	8.01599400	6.05115900
C	0	5.02380900	5.10695000	6.16976900
C	-1	4.07450500	5.15498400	5.14692700
H	0	2.40172400	6.25594800	4.28309700
H	0	5.75644300	4.29178500	6.20380800
H	0	4.05994000	4.36095200	4.39101600
C	0	11.35947800	7.28024700	14.27604000
C	0	11.27752500	6.22381800	15.21459500
C	0	12.40588900	7.24463200	13.32330000
C	0	12.20689300	5.17506800	15.20510100
H	0	10.48381300	6.23632200	15.97005500
C	0	13.33159400	6.19109400	13.31222700
H	0	12.48289900	8.03763700	12.57203300
C	-1	13.23820300	5.15374500	14.25307000
H	0	12.12812500	4.37331800	15.94825000
H	0	13.96649200	4.33499200	14.24505200
C	0	11.13261500	14.50030600	7.04821000
C	0	12.25995300	15.35461900	7.09749800
C	0	11.08058300	13.52885300	6.01919900

C	0	13.29089000	15.24615100	6.15344500
H	0	12.31846600	16.12263200	7.87687300
C	0	12.11572900	13.41864100	5.07954600
H	0	10.23060700	12.84056100	5.96490900
C	-1	13.22464500	14.27744100	5.13923500
H	0	14.14829700	15.92669600	6.20885200
H	0	12.05703500	12.65126100	4.29914000
H	0	14.02988300	14.19401800	4.40081100
H	0	8.71009900	6.94077500	14.42318700
H	0	3.83317300	11.64677500	8.50092600
H	0	9.78538100	14.82998900	11.51678400
H	0	14.12662700	6.17925300	12.55781700
N	0	11.98230000	10.18027100	14.08927200
H	0	12.15494200	11.15187800	14.35094600
H	0	12.71168400	9.54750500	14.42122100
N	0	8.23364200	14.51627100	6.43420000
H	0	7.27630300	14.82703200	6.26383900
H	0	8.88313400	14.81980400	5.70718600
N	0	6.10437900	6.07703100	8.10072800
H	0	6.02387100	6.64734500	8.93987500
H	0	6.57838100	5.18559900	8.24233500

10-mNH$_2^{4+}$ (triplet)

E = -14095.31948710 H

Zr	0	6.30560300	14.54305200	14.32912500
O	0	4.86106000	13.58276900	15.61849600
O	0	5.08822500	13.32071900	13.11917400
O	0	7.42337100	14.84303400	12.37678700
C	0	7.01373100	14.81569300	11.16553300
C	0	8.05839000	14.75090700	10.09098400
C	0	7.68026300	14.69233700	8.73991200
C	0	6.58845500	15.79589300	16.67966600
C	0	8.55715500	15.45646800	15.54808700

H	0	9.50756400	15.02548800	15.22013900
C	0	7.74078400	14.95100500	16.60860300
C	0	10.02816900	14.59392300	8.04604600
C	0	8.63707100	14.61372500	7.70080600
O	0	5.78705400	14.85128300	10.81144100
C	0	9.42806600	14.74819000	10.43002000
C	0	10.38143100	14.67656400	9.41117900
C	0	6.69583300	16.80630700	15.68020700
C	0	7.92086200	16.59907800	14.98357400
C	0	2.72282800	11.75776900	16.72141400
H	0	2.84624500	12.59365000	17.41460700
C	0	3.04055600	9.78205900	15.58825800
H	0	3.46694100	8.83127500	15.25554100
C	0	3.56685700	10.61715300	16.61735600
C	0	2.26517200	16.61470800	11.74376800
H	0	2.28761700	17.30106100	12.59447100
C	0	2.69540700	15.63830200	9.70761600
H	0	3.14059400	15.42062700	8.73253000
C	0	3.05554100	16.72756300	10.55848800
C	0	1.67598200	11.62073400	15.76227700
H	0	0.85822800	12.32971700	15.61320100
C	0	1.85351600	10.38241600	15.06734900
C	0	1.42448500	15.46772700	11.61838200
H	0	0.68132400	15.13748200	12.34746600
C	0	1.69179700	14.86462300	10.35764800
Zr	0	3.83014100	14.51226900	11.94776800
O	0	4.73814500	15.82469900	13.49971900
O	0	3.57692200	12.58242800	10.86340000
C	0	3.67453600	11.33292800	11.21009200
C	0	3.82016600	10.32095100	10.17554300
C	0	3.92690200	8.93009200	10.51134300
C	0	4.07841500	8.29150700	8.12858700
C	0	4.05199200	7.95914800	9.53575000
O	0	3.61503400	10.94190300	12.45105100

C	0	3.83014800	10.66213200	8.77871400
C	0	3.93379000	9.69132500	7.80246400
Zr	0	3.92615200	12.04224200	14.35096900
O	0	2.60286500	13.58681200	13.54763400
O	0	5.94026700	10.90118900	14.46401000
C	0	7.14489200	11.31359600	14.40078000
C	0	8.22572600	10.27324400	14.33061900
C	0	9.57111200	10.66083600	14.23060900
C	0	10.28698900	8.31959200	14.25560000
C	0	10.61914000	9.71179000	14.18535400
O	0	7.49499300	12.53882200	14.41590500
C	0	7.89834900	8.90273700	14.39234500
C	0	8.92615600	7.95697700	14.36248600
C	0	3.37813500	19.05333400	9.57060800
H	0	2.85486200	18.72509600	8.65085800
H	0	2.59625800	19.45432600	10.24546200
C	0	4.37529100	20.16908400	9.22244200
H	0	5.15075600	19.76519500	8.54162100
H	0	4.90796900	20.48123500	10.14277900
C	0	3.71513200	21.39243100	8.57614100
H	0	4.45980900	22.17455800	8.34045200
H	0	3.20424400	21.12248400	7.63280900
H	0	2.95892700	21.84193300	9.24692500
C	0	8.05470400	13.79271200	17.52319300
H	0	7.12076100	13.24715300	17.75951400
H	0	8.71445000	13.07755300	16.99893300
C	0	8.72808600	14.22361200	18.84521600
H	0	9.66877800	14.76114400	18.61428600
H	0	8.07412600	14.95122700	19.36509800
C	0	9.02779800	13.04305300	19.78179400
H	0	8.08313400	12.50824100	20.00429700
H	0	9.67395500	12.31510400	19.25230300
C	0	9.69909000	13.46021200	21.09544800
H	0	9.90086500	12.58431700	21.73889600

H	0	10.66395300	13.96910600	20.91116800
H	0	9.06028600	14.15657500	21.67071900
C	0	0.93641000	9.77363100	14.02993100
H	0	1.54021800	9.17641800	13.32198800
H	0	0.26764700	9.04737100	14.53718300
C	0	0.06893800	10.77475300	13.24959500
H	0	-0.57813300	11.33186600	13.95468200
H	0	0.72274400	11.52601800	12.76535200
C	0	-0.81207200	10.10855700	12.18115000
H	0	-0.16494800	9.57094300	11.46015300
H	0	-1.44551000	9.33609100	12.66060300
C	0	-1.70268200	11.10102300	11.42518500
H	0	-2.39650100	11.62208500	12.11148100
H	0	-1.09844600	11.87380300	10.91322800
H	0	-2.31283300	10.59170900	10.65694600
H	0	1.22451400	13.95717400	9.96813100
H	0	5.76167700	15.68657600	17.38706200
H	0	8.29549800	17.19575600	14.14775100
H	0	5.97433700	17.60609100	15.49521100
H	0	4.47106800	10.42678200	17.20150100
H	0	3.88285200	8.63527200	11.56419600
H	0	3.95922100	9.99847200	6.75152800
H	0	4.05757800	6.90873500	9.84521500
H	0	9.82042300	11.72649600	14.19640200
H	0	6.61710600	14.70996700	8.47856100
H	0	11.44488000	14.65714400	9.67445900
C	0	4.03972700	17.82616200	10.23689900
H	0	4.82596800	17.43251200	9.56649900
H	0	4.54813700	18.15261000	11.16395700
H	0	1.64015700	13.45792700	13.47770200
H	0	4.97622000	13.48592000	16.58127200
H	0	6.85254000	8.59158500	14.46452900
H	0	4.85475500	16.78616600	13.38909100
C	0	4.17321900	7.29047600	7.09095500

C	0	3.55852100	7.46298600	5.81524400
C	0	4.89031700	6.01540800	7.27962200
C	0	3.49836900	6.43349900	4.88099800
H	0	3.04652000	8.40565300	5.60044000
C	0	4.76363300	4.95697000	6.33347600
C	-1	4.07450500	5.15498400	5.14692700
H	0	2.96845800	6.59862900	3.93636300
H	0	5.27889400	4.01217600	6.53983200
H	0	3.99791600	4.35128500	4.40835200
C	0	11.32587300	7.25001800	14.25338900
C	0	11.25926100	6.20168400	15.20207700
C	0	12.37240800	7.22013100	13.30044800
C	0	12.20536200	5.16783900	15.20365100
H	0	10.46527400	6.20976000	15.95732700
C	0	13.31490700	6.18167500	13.30054900
H	0	12.43730400	8.00681300	12.54143800
C	-1	13.23820300	5.15374500	14.25307000
H	0	12.13902800	4.37258400	15.95496700
H	0	13.97990000	4.34705600	14.25464100
C	0	11.10562200	14.49860700	7.01854800
C	0	12.23889200	15.34353500	7.09256200
C	0	11.06005300	13.53964000	5.97753800
C	0	13.28328700	15.23580100	6.16359600
H	0	12.29226700	16.10297000	7.88065100
C	0	12.10861100	13.43018500	5.05271000
H	0	10.20517200	12.85935700	5.90326500
C	-1	13.22464500	14.27744100	5.13923500
H	0	14.14558600	15.90817700	6.23901300
H	0	12.05503000	12.67166500	4.26330000
H	0	14.04068300	14.19364100	4.41279700
H	0	8.67534100	6.89109300	14.40488700
H	0	3.75823200	11.71628100	8.49223100
H	0	9.73360100	14.79308200	11.47888600
H	0	14.11040000	6.17450400	12.54656000

N	0	11.92673900	10.15008600	14.04689700
H	0	12.09209100	11.12405500	14.30457900
H	0	12.66045300	9.52457400	14.38308800
N	0	8.20700900	14.52238300	6.38665600
H	0	7.25087400	14.83606100	6.21455200
H	0	8.86016600	14.83516300	5.66679900
N	0	5.74104600	5.86883800	8.31881300
H	0	6.26776000	5.00080800	8.43823600
H	0	5.98325700	6.65455900	8.92461200

1-NH_2^{4+} (singlet)

E = -13402.57953840 H

Zr	0	6.35417000	14.45877800	14.42677500
O	0	4.83704800	13.52005600	15.67869500
O	0	5.11478900	13.26963300	13.19538300
O	0	7.48093500	14.75464500	12.50042800
C	0	7.14079200	14.62477300	11.26290800
C	0	8.21995500	14.51217500	10.25024700
C	0	7.85758100	14.44844600	8.88056500
C	0	6.62063500	15.70526700	16.78572300
C	0	8.59399600	15.36215400	15.66434100
H	0	9.54774800	14.93134000	15.34584600
C	0	7.77131800	14.85861000	16.72107000
C	-1	10.17619900	14.39080000	8.21230000
C	0	8.80681900	14.38867400	7.86366100
O	0	5.92548300	14.61948600	10.87124600
C	0	9.61656900	14.49161900	10.60283300
C	0	10.57067000	14.43632800	9.54403500
C	0	6.73723200	16.71827900	15.78924700
C	0	7.96533200	16.50911300	15.09878400
C	0	2.71773300	11.71729500	16.71680500
H	0	2.88115000	12.53690100	17.42047500
C	0	2.93337600	9.73421400	15.56765800

H	0	3.31553900	8.77042500	15.21957600
C	0	3.51302200	10.54352000	16.58719000
C	0	2.63969800	16.83691000	11.73928700
H	0	2.73625900	17.54187700	12.56967200
C	0	3.00394100	15.71750300	9.76830800
H	0	3.44113800	15.40346500	8.81592700
C	0	3.49313900	16.77991900	10.59317500
C	0	1.66091200	11.63761100	15.76305900
H	0	0.87553800	12.38422000	15.62471900
C	0	1.77990000	10.40020300	15.05503700
C	0	1.64016900	15.82782200	11.62072400
H	0	0.83689700	15.63187700	12.33553800
C	0	1.86168500	15.13877300	10.39355000
Zr	0	3.90841600	14.48646100	12.00945900
O	0	4.80151300	15.75679400	13.56733000
O	0	3.62073100	12.53130000	10.88704200
C	0	3.66612300	11.28752700	11.22071200
C	0	3.77790700	10.26651400	10.15027000
C	0	3.85268500	8.89913400	10.52296800
C	-1	3.99770100	8.21230100	8.21230000
C	0	3.95987100	7.87592200	9.58490700
O	0	3.59557900	10.88496900	12.43792300
C	0	3.81765700	10.60633100	8.75004900
C	0	3.93040100	9.54015800	7.80673700
Zr	0	3.93135600	11.99862900	14.38693200
O	0	2.63070800	13.57480900	13.55634500
O	0	5.89869100	10.85370200	14.63206100
C	0	7.11508100	11.23423400	14.54568800
C	0	8.18058000	10.17952500	14.47127000
C	0	9.50181300	10.54122100	14.13042500
C	-1	10.17620000	8.21230000	14.39080100
C	0	10.48619000	9.54393700	14.09083400
O	0	7.49820700	12.44899700	14.53771400
C	0	7.85861500	8.84153100	14.75894600

C	0	8.85070500	7.83258800	14.72657500
C	0	4.19585300	19.02244400	9.60431400
H	0	3.67028800	18.77898200	8.66005400
H	0	3.45452100	19.53359000	10.24967800
C	0	5.36421300	19.97599300	9.31058400
H	0	6.10639900	19.45516900	8.67363400
H	0	5.88672000	20.21195200	10.25885100
C	0	4.93211400	21.27936200	8.62879700
H	0	5.79914900	21.93652800	8.43307800
H	0	4.43665100	21.08211100	7.65951900
H	0	4.21934400	21.84551300	9.25756400
C	0	8.08279800	13.70071300	17.63683000
H	0	7.14700300	13.16320000	17.88299800
H	0	8.73360700	12.97949000	17.10964700
C	0	8.76996100	14.13218800	18.95162100
H	0	9.71216900	14.66260300	18.71074500
H	0	8.12493800	14.86589300	19.47405600
C	0	9.06988800	12.95309700	19.88994500
H	0	8.12388800	12.42464700	20.12174500
H	0	9.70779400	12.21966300	19.35806000
C	0	9.75353000	13.37113700	21.19688600
H	0	9.95512900	12.49638500	21.84193500
H	0	10.71989200	13.87374300	21.00348000
H	0	9.12306300	14.07321400	21.77441100
C	0	0.82791800	9.84132300	14.02162700
H	0	1.38816100	9.17204700	13.34276200
H	0	0.09460700	9.19058700	14.54230700
C	0	0.05594900	10.88268400	13.19418400
H	0	-0.54170100	11.52610800	13.86882000
H	0	0.77631000	11.55010300	12.68287200
C	0	-0.87601300	10.24747900	12.15019400
H	0	-0.28448400	9.57433500	11.49846000
H	0	-1.61498700	9.60307800	12.66625500
C	0	-1.61044800	11.27571600	11.28270300

H	0	-2.23918500	11.94741500	11.89711000
H	0	-0.89742200	11.90821600	10.72042700
H	0	-2.27070300	10.78232600	10.54623700
H	0	1.27646800	14.29886000	10.01008700
H	0	5.79042300	15.59800000	17.48941100
H	0	8.34665600	17.10643200	14.26657200
H	0	6.01848100	17.51985500	15.60124600
H	0	4.40772900	10.30542600	17.16800000
H	0	3.82893600	8.66389400	11.59143100
H	0	3.96184000	9.79584500	6.74126700
H	0	4.01633100	6.83059100	9.90550000
H	0	9.73909000	11.58142700	13.88913000
H	0	11.51724100	9.80526500	13.82588400
H	0	6.78989400	14.45000200	8.63908100
H	0	8.49800500	14.34494900	6.81417400
H	0	11.63504700	14.43010000	9.80624100
H	0	4.08255100	7.42422200	7.45489200
H	0	10.95951100	7.44559900	14.36412100
H	0	10.94185200	14.35310500	7.42841500
C	0	4.64110800	17.71003700	10.28745900
H	0	5.37032200	17.19452300	9.63631000
H	0	5.17363200	17.95893000	11.22542000
H	0	1.66486500	13.48414600	13.46345200
H	0	4.93648000	13.41322800	16.64232400
H	0	6.82833000	8.57929200	15.02171600
N	0	3.73601100	11.88599600	8.29894700
H	0	3.85411000	12.08277600	7.30773500
H	0	3.73625600	12.64351200	8.98270900
N	0	10.05820900	14.49691400	11.89230100
H	0	11.04961700	14.64041600	12.07436900
H	0	9.37614900	14.68782000	12.62773400
N	0	8.52634000	6.50724500	14.95923500
H	0	9.29422800	5.89513600	15.23731200
H	0	7.66963900	6.33610500	15.48683200

H	0	4.92390100	16.71887600	13.46858500

1-NH$_2^{4+}$ (triplet)

E = -13402.48625420 H

Zr	0	6.39106000	14.38969800	14.51484200
O	0	4.81074600	13.55521300	15.77932900
O	0	5.15940700	13.18628900	13.30066600
O	0	7.54329700	14.56389400	12.54661300
C	0	7.18849200	14.52157700	11.31072700
C	0	8.25223600	14.44312600	10.27908000
C	0	7.86879500	14.41679100	8.91543000
C	0	6.69923200	15.75600100	16.79406400
C	0	8.64228500	15.22487900	15.69385000
H	0	9.56582900	14.71756100	15.40089800
C	0	7.79572400	14.83800600	16.77841600
C	-1	10.17619700	14.39079700	8.21230100
C	0	8.80282000	14.38887300	7.88281200
O	0	5.97007000	14.57624900	10.93033900
C	0	9.65524700	14.42295900	10.61076100
C	0	10.59353200	14.40434400	9.53696300
C	0	6.86913200	16.68847300	15.73090300
C	0	8.07429300	16.36025100	15.04756100
C	0	2.65706800	11.68216800	16.81017900
H	0	2.82407300	12.48201400	17.53575700
C	0	2.86329200	9.72454700	15.61399600
H	0	3.23156800	8.75926700	15.25519000
C	0	3.43381000	10.49799800	16.66625900
C	0	2.71053700	16.80158200	11.80885800
H	0	2.81108700	17.50232600	12.64239300
C	0	3.07423000	15.68224500	9.83793900
H	0	3.51108900	15.37016800	8.88468700
C	0	3.56525000	16.74282700	10.66491600
C	0	1.62456400	11.64761700	15.82872100

H	0	0.85774500	12.41350600	15.68964500
C	0	1.74090500	10.43045900	15.08508100
C	0	1.70645300	15.79762300	11.68608300
H	0	0.90129500	15.60410700	12.39940500
C	0	1.92459700	15.11135700	10.45691100
Zr	0	3.96940100	14.43452900	12.07748400
O	0	4.90522800	15.71140700	13.61906700
O	0	3.65956200	12.54286000	10.98498900
C	0	3.71216400	11.26969100	11.32596000
C	0	3.83021400	10.26282500	10.28753200
C	0	3.86750500	8.87611000	10.59663400
C	-1	3.99770400	8.21230300	8.21230100
C	0	3.94446000	7.88899100	9.59193000
O	0	3.62230200	10.89097000	12.57301300
C	0	3.90914900	10.58906600	8.84534700
C	0	3.98494600	9.56970400	7.85667600
Zr	0	3.93308400	11.98372500	14.48927900
O	0	2.65652600	13.58965400	13.62768400
O	0	5.89487200	10.85365200	14.76284200
C	0	7.11276600	11.22797400	14.69784300
C	0	8.17773600	10.17952400	14.57471400
C	0	9.50322000	10.55492900	14.26237600
C	-1	10.17619900	8.21230100	14.39079800
C	0	10.48653500	9.55977800	14.17054900
O	0	7.49870400	12.44768300	14.75228000
C	0	7.85493100	8.82643400	14.78284900
C	0	8.84631200	7.81913200	14.69781700
C	0	4.28226000	18.97717100	9.66203800
H	0	3.77836500	18.72739100	8.70761100
H	0	3.52562200	19.48994500	10.28810900
C	0	5.45594700	19.93104500	9.39016200
H	0	6.21092100	19.40943700	8.76890600
H	0	5.95943700	20.16628800	10.34880300
C	0	5.04015400	21.23539600	8.70056500

H	0	5.91254200	21.89061200	8.52288100
H	0	4.56375400	21.03966000	7.72150900
H	0	4.31581100	21.80293300	9.31472700
C	0	8.04576900	13.72524700	17.76620100
H	0	7.08172300	13.26482900	18.05546800
H	0	8.64353800	12.93221900	17.28130000
C	0	8.77765600	14.19331300	19.04386000
H	0	9.74522500	14.65150800	18.75916500
H	0	8.18590100	14.99338800	19.53071400
C	0	9.02400800	13.05433000	20.04518700
H	0	8.05181700	12.60447700	20.32860800
H	0	9.59880000	12.24986100	19.54493000
C	0	9.76730300	13.50056700	21.30945300
H	0	9.91831000	12.65502000	22.00536400
H	0	10.76367900	13.91437500	21.06506700
H	0	9.20629400	14.28442700	21.85241400
C	0	0.79455400	9.90439500	14.02938900
H	0	1.34980600	9.22083300	13.36055100
H	0	0.03241300	9.27356900	14.53354900
C	0	0.06295700	10.96248900	13.18650500
H	0	-0.52748300	11.62384000	13.85020500
H	0	0.80695100	11.60761600	12.68075300
C	0	-0.87178700	10.34055500	12.13676900
H	0	-0.28766700	9.65181600	11.49440400
H	0	-1.62695800	9.71324100	12.65057400
C	0	-1.58161500	11.37327500	11.25528000
H	0	-2.20749400	12.05844000	11.85760400
H	0	-0.85542200	11.99078200	10.69344500
H	0	-2.24303900	10.88315500	10.51752100
H	0	1.33098500	14.27998000	10.06822800
H	0	5.86576000	15.74028500	17.50149300
H	0	8.48652000	16.88019100	14.17937500
H	0	6.19660200	17.51597700	15.49144100
H	0	4.30980200	10.22683800	17.26147000

H	0	3.83207900	8.57318100	11.64658200
H	0	4.03756400	9.88153900	6.80750000
H	0	3.96379000	6.83605400	9.89728100
H	0	9.74573600	11.60590800	14.08099000
H	0	11.51877900	9.83638300	13.92625200
H	0	6.79764300	14.42054600	8.69045100
H	0	8.47911500	14.36986200	6.83713100
H	0	11.66201400	14.39834100	9.78116900
H	0	4.05523500	7.42994100	7.45112900
H	0	10.95790300	7.44667400	14.32462700
H	0	10.92981900	14.37758500	7.41585100
C	0	4.71652300	17.67009900	10.36220600
H	0	5.45405200	17.14909700	9.72479600
H	0	5.23876800	17.92885700	11.30349700
H	0	1.69830900	13.48083400	13.49069500
H	0	4.90924200	13.46730000	16.74485200
H	0	6.82302000	8.55011700	15.02460800
N	0	3.91641500	11.88270000	8.47201500
H	0	3.97279000	12.14698400	7.48558500
H	0	3.84794800	12.58579200	9.23230400
N	0	10.11277000	14.40936300	11.89095400
H	0	11.11075000	14.48458800	12.07448600
H	0	9.43284200	14.49111000	12.64815000
N	0	8.51793700	6.48468200	14.85481700
H	0	9.28166600	5.85077300	15.09133300
H	0	7.65518000	6.28158500	15.36025600
H	0	5.05601200	16.66475500	13.48566200

S9. References

[1]Gaussian 09, Revision D.01, M. J. Frisch; G. W. Trucks, H. B. Schlegel, G. E. Scuseria, M. A. Robb, J. R. Cheeseman, G. Scalmani, V. Barone, B. Mennucci, G. A. Petersson, H. Nakatsuji, M. Caricato, X. Li, H. P. Hratchian, A. F. Izmaylov, J. Bloino, G. Zheng, J. L. Sonnenberg, M. Hada, M. Ehara, K. Toyota, R. Fukuda, J. Hasegawa, M. Ishida, T. Nakajima, Y. Honda, O. Kitao, H. Nakai, T. Vreven, J. A. Montgomery, Jr., J. E. Peralta, F. Ogliaro, M. Bearpark, J. J. Heyd, E. Brothers, K. N. Kudin, V. N. Staroverov, R. Kobayashi, J. Normand, K. Raghavachari, A. Rendell, J. C. Burant, S. S. Iyengar, J. Tomasi, M. Cossi, N. Rega, J. M. Millam, M. Klene, J. E. Knox, J. B. Cross, V. Bakken, C. Adamo, J. Jaramillo, R. Gomperts, R. E. Stratmann, O. Yazyev, A. J. Austin, R. Cammi, C. Pomelli, J. W. Ochterski, R. L. Martin, K. Morokuma, V. G. Zakrzewski, G. A. Voth, P. Salvador, J. J. Dannenberg, S. Dapprich, A. D. Daniels, Ö. Farkas, J. B. Foresman, J. V. Ortiz, J. Cioslowski, and D. J. Fox, Gaussian, Inc., Wallingford CT, 2009.

[2]Tao, J.; Perdew, J. P.; Staroverov, V. N.; Scuseria, G. E. Climbing the Density Functional Ladder: Nonempirical Meta--Generalized Gradient Approximation Designed for Molecules and Solids. *Phys. Rev. Lett.***2003**, *91* (14), 146401

[3] Andrae, D., Häußermann, U., Dolg, M., Stoll, H., Preuß, H. Energy-adjustedab initio pseudopotentials for the second and third row transition elements. *Theor. Chim. Acta*, **1990**, *77*, 123-141

[4] Weigend, F., Ahlrichs, R. Balanced basis sets of split valence, triple zeta valence and quadruple zeta valence quality for H to Rn: Design and assessment of accuracy. *Phys. Chem. Chem. Phys*., **2005**, *7*, 3297

[5]Marenich, A. V.; Cramer, C. J.; Truhlar, D. G. Universal Solvation Model Based on Solute Electron Density and on a Continuum Model of the Solvent Defined by the Bulk Dielectric Constant and Atomic Surface Tensions. *J. Phys. Chem. B* **2009**, *113* (18), 6378–6396.

[6] Álvarez-Moreno, M.; de Graaf, C.; Lopez, N.; Maseras, F.; Poblet, J.M.; Bo, C. *J. Chem. Inf. Model*. **2015**, *55*, 95-103.

[7] Delgado, P.; Martin-Romera, J. D.; Perona, C.; Vismara, R.; Galli, S.; Maldonado, C. R.; Carmona, F. J.; Padial, N. M.; Navarro, J. A. R. *ACS Appl. Mater*. **2022**, *14*, 26501–26506.

[8] P. J. Jabalera-Ortiz, C. Perona, M. Moreno-Albarracín, F. J. Carmona, J.-R. Jiménez, J. A. R. Navarro, P. Garrido-Barros, *Angew. Chem. Int. Ed.* 2024, e202411867.

[9] Espinoza, E. M.; Clark, J. A.; Soliman, J.; Derr, J. B.; Morales, M.; Vullev, V. I. **2019** *J. Electrochem. Soc.* **166** H3175

[10] Robert H. Blessin Acta Cryst. (1995). As1, 33-38

[11] Sheldrick, G. M. SHELXT-2014, 2013, Sheldrick, G.M. (2015). Acta Cryst. C71, 3-8

[1212] Dolomanov, O. V.; Bourhis, L. J.; Gildea, R. J.; Howard, J. A. K.; Puschmann, H. J. Appl. Crystallogr. 2009, 42, 339-341.

[13] Spek, A. L. Acta Cryst. (2015). C71, 9–18.

[14] Rabani, J.; Mamane, H.; Pousty, D.; Bolton, J. R. Practical Chemical Actinometry—A Review. *Photochem. Photobiol*. **2021**, *97*, 873–902.
